# Prediction of bacteria-specific drug metabolism using a cross-attention-based model

**DOI:** 10.1093/bib/bbag578

**Published:** 2026-10-19

**Authors:** Jaehyeon Park, Si Eun Kim, Heeyoung Yang, Nam-Chul Cho, Hyunho Kim, Hyun Kil Shin

**Affiliations:** Drug Information Platform Center, Chemical Platform Technology Division, Korea Research Institute of Chemical Technology (KRICT), 141, Gajeong-ro, Yuseong-gu, Daejeon 34114, Republic of Korea; Department of Medicinal Chemistry and Pharmacology, University of Science and Technology (UST), 217, Gajeong-ro, Yuseong-gu, Daejeon 34113, Republic of Korea; Chemical Analysis Center, Chemical Platform Technology Division, Korea Research Institute of Chemical Technology (KRICT), 141, Gajeong-ro, Yuseong-gu, Daejeon 34114, Republic of Korea; Center for Biomimetic Research, Korea Institute of Toxicology (KIT), 141, Gajeong-ro, Yuseong-gu, Daejeon 34114, Republic of Korea; Drug Information Platform Center, Chemical Platform Technology Division, Korea Research Institute of Chemical Technology (KRICT), 141, Gajeong-ro, Yuseong-gu, Daejeon 34114, Republic of Korea; Department of Medicinal Chemistry and Pharmacology, University of Science and Technology (UST), 217, Gajeong-ro, Yuseong-gu, Daejeon 34113, Republic of Korea; School of Artificial Intelligence, Chonnam National University, 77 Yongbong-ro, Buk-gu, Jeonnam Gwangju 61186, Republic of Korea; Department of AI-Based Drug Discovery, Duksung Women’s University, 33, Samyang-ro 144-gil, Dobonggu Seoul 01369, Republic of Korea

**Keywords:** bacteria-specific drug metabolism, drug-microbe interaction, bidirectional cross-attention, gut microbiome, personalized medicine, interpretability

## Abstract

Drug metabolism by gut microorganisms is bacteria-specific and can have clinically significant consequences, including prodrug activation, drug inactivation, and the generation of toxic metabolites. However, existing computational approaches have primarily focused on community-level predictions or drug-to-microbe effects, without resolving the metabolic contributions of individual bacterial strains or the bidirectional nature of drug–bacteria interactions, despite advances in computational frameworks. Here, we propose a bidirectional cross-attention architecture, multimodal Bi-MHA, that integrates atom-level molecular embeddings and protein-level bacterial embeddings from pretrained models to generate a combined representation for classification. The proposed model was developed using reliability stratification and a $\mathrm{\lambda}$-scaled loss function on the training dataset and achieved consistent performance across three unseen tasks: unseen drug (receiver operating characteristic area under the curve [ROC AUC]: 0.690), unseen bacteria (ROC AUC: 0.920), and unseen interaction (ROC AUC: 0.949), ranking first among all compared models in the rank-sum metric. Beyond prediction, attention-based analysis not only indicated enzymatic reaction sites that were experimentally validated in previous studies but also prioritized candidate bacterial proteins, demonstrating interpretability at both the atom and protein levels. The proposed model also achieved a 15 percentage point improvement in accuracy over the KNN baseline on an external validation dataset of 85 out-of-distribution drug-bacteria pairs. Furthermore, the model was extended to community-level prediction as a proof-of-concept application using a weighted-abundance pipeline on an independent microbiome dataset. These results demonstrate the model’s potential as an interpretable screening tool for drug safety evaluation and personalized medicine. The source code is available at https://github.com/Jaehyeon-O-Ob/Bacteria-Specific_Drug_Metabolism.

## Introduction

Orally administered drugs are metabolized by gut microorganisms. Gut microorganisms induce structural and functional transformations of drugs through various metabolic reactions, including reduction and hydrolysis [1]. This microbial drug metabolism is clinically important for several reasons. First, some drugs are administered as inactive prodrugs and are converted into active agents through metabolism by gut microorganisms. For example, sulfasalazine, which is used to treat ulcerative colitis and Crohn’s disease, is metabolized into 5-aminosalicylic acid (5-ASA), the active therapeutic agent in the colon [2, 3]. Second, microbial metabolism can reduce drug efficacy. A notable example is the inactivation of digoxin by *Eggerthella lenta*; in ~10% of patients, this specific gut microorganism reduces digoxin to its inactive form, dihydrodigoxin [4–6]. Third, metabolites generated through microbial metabolism can cause unexpected adverse effects. CPT-11, an anticancer agent that inhibits DNA topoisomerase I, is hydrolyzed by carboxylesterase to the active metabolite SN-38, which causes intestinal damage and diarrhea [7]. For these reasons, gut microorganism-mediated drug metabolism should be more actively considered in drug safety assessment, toxicity prediction, and personalized dosing strategies [1, 8–11]. Moreover, these metabolic processes indicate that a protein-level approach is essential because microbiome-encoded enzymes are directly involved, and related studies have been conducted at the protein level [1, 8, 10].

However, *in vitro* experimental approaches for studying gut microorganism-mediated drug metabolism at the protein level face substantial time and cost constraints. In particular, anaerobic culture systems that recapitulate the intestinal environment present several technical challenges in experimental design [12, 13]. Computational methods can help address these experimental limitations. Indeed, the applicability of new approach methodologies (NAMs), including *in silico* approaches, is being explored for studying microbiome–host interactions and understanding the effects of gut microbial metabolic functions on the host [14–16].

Many *in silico* studies of microbe–drug interactions incorporate both drug representations and microbial information, recognizing that both modalities are essential for capturing the complex relationship between drugs and microbes. Algavi et al. developed a machine learning framework that integrates chemical and genomic data, representing each drug with 92 physicochemical properties derived from Simplified Molecular Input Line Entry System (SMILES) and each microbe with 148 genomic features derived from Kyoto Encyclopedia of Genes and Genomes (KEGG) pathway abundances [17]. Similarly, Long et al. proposed GCNMDA, a graph convolutional network framework that constructs a heterogeneous network incorporating drug chemical similarity and microbe genomic similarity, learning joint representations through graph-based message passing to predict microbe–drug associations [18]. Deng et al. further developed Graph2MDA, a variational graph autoencoder model that integrates multiple data modalities, including molecular structures, microbial genetic sequences, and functional annotations, to learn enriched latent representations for microbe–drug prediction [19]. Collectively, these findings demonstrate that combining drug-specific and microbe-specific representations is essential for accurately predicting complex relationships between drugs and microbes.

Although these studies established a foundation for computational analyses of microbe–drug interactions using multimodal approaches, they inherently rely on conventional descriptors, such as physicochemical properties, or hand-crafted fingerprints, such as Morgan fingerprints, which may not fully capture complex structural and functional relationships. Recent advances in deep learning have introduced more sophisticated representation learning methods. In the molecular domain, Hu et al. explored strategies for pretraining graph neural networks (GNNs), demonstrating that graph isomorphism networks (GINs) pretrained with self-supervised tasks on large-scale unlabeled molecular datasets significantly improve downstream molecular property prediction [20]. Fang et al. developed GEM, a geometry-enhanced GNN framework that incorporates 3D spatial information, including bond angles, into the molecular graph, demonstrating that the pretrained representations achieved an average improvement of 8.8% over existing GNN baselines in molecular property prediction [21]. Similarly, in the protein domain, Lin et al. introduced Evolutionary Scale Modeling (ESM)-2, a protein language model with up to 15 billion parameters trained on millions of protein sequences using masked language modeling. This model learns evolutionary and structural patterns directly from sequences and enables accurate protein structure prediction [22]. Notably, Elnaggar et al. demonstrated that embeddings derived from pretrained protein language models consistently outperform conventional hand-crafted descriptors, such as amino acid composition and k-mer representations, across multiple downstream tasks, including secondary structure prediction [23]. These pretrained foundation models for molecules and proteins offer several critical advantages, including contextual representations beyond the capacity of hand-crafted descriptors, effective transfer learning to downstream tasks, and rich, generalizable embeddings for diverse prediction tasks.

Leveraging these advances, substantial research has focused on predicting the effects of drugs on microbial systems, introducing effective computational approaches that use deep learning and pretrained molecular representations for the discovery of novel antimicrobial compounds [15, 24]. However, the reverse direction, chemical transformation of drugs by gut microbes, has received comparatively less attention. McCoubrey et al. developed a classification model that predicts drug susceptibility to microbiota-mediated depletion, a process that can affect drug exposure, achieving an ROC AUC of 0.91 [16]. Nevertheless, these models operate at the gut microbiome community level without resolving which specific bacterial species contribute to the metabolic transformation of individual drugs.

To address these challenges, we developed the multimodal Bi-MHA model, a bidirectional cross-attention framework that predicts drug metabolic state at the individual bacterial strain level and provides mechanistic interpretability through atom- and protein-level attention analysis. This approach makes the following contributions:

(1) Novel *in silico* framework for bacteria-specific drug metabolism: Our model enables early-stage safety screening by predicting the metabolic state of drugs in specific gut bacteria, helping to address experimental limitations and culture instability.
(2) Atom-level and protein-level interpretable model: We design a predictive model based on a cross-attention mechanism to provide insights into which molecular substructures and which microbial proteins the model associates with a predicted metabolic interaction. The model uses protein sequences to generate microbial representations, thereby resolving these interpretations at the level of individual microbial strains.
(3) Reliability-stratified training with $\mathrm{\lambda}$-scaled loss: We introduce a training strategy that assigns reliability-based weights $\mathrm{\lambda}$ to each sample’s loss.
(4) Community-level metabolic prediction: We extend the strain-level predictions to microbial community-level outcomes as a proof-of-concept through a weighted-abundance pipeline, bridging individual species predictions to gut microbiome contexts.

## Materials and methods

Our approach for developing the predictive model consists of several steps, from representation generation to classification.

### Molecular representation

To represent the chemical properties of drugs, we constructed three molecular representations from SMILES strings. First, 217 physicochemical descriptors were calculated to capture global molecular properties using the complete standard descriptor set provided by RDKit Python library (version 2025.3.1) [25] without feature selection. Second, Extended-Connectivity Fingerprints (ECFP4) with 1024 bits were generated to encode substructural motifs. Both representations were computed using the RDKit Python library (version 2025.3.1) [25]. Third, MolE, a pretrained GIN-based model [24], was used to extract atom-level embeddings for each molecule. From these embeddings, a mean-pooled vector was derived as a fixed-length representation for traditional models and the multilayer perceptron (MLP), whereas the full set of atom-level token embeddings was used as sequential input to the cross-attention module, enabling atom-level interpretability. Detailed descriptions are provided in the Supplementary Methods, *Molecular Representation* section, and the interpretability analysis is presented in the *Case Study* section.

### Bacterial representation

To represent the 71 gut bacterial strains, we constructed four distinct bacterial representations derived from strain names and protein sequences. Detailed descriptions are provided in the Supplementary Methods, *Bacterial Representation* section.

First, a one-hot encoded vector based on taxonomic identity served as the baseline representation. Second, Composition, Transition, and Distribution (CTD) descriptors [26, 27] were computed for each protein and aggregated by mean pooling to produce a strain-level physicochemical representation. Third, a KEGG pathway-based representation [28] was constructed by assigning protein sequences to KEGG Orthology IDs using KofamScan [29], yielding a 204-dimensional pathway score vector for each strain that encodes functional metabolic capacity. Fourth, ESM-C 600M [30, 31], a pretrained protein language model, was used to extract context-rich protein embeddings. Proteins were filtered to retain only those assigned to metabolism-related KEGG pathways to reduce noise. For each selected protein, ESM-C produced residue-level embeddings that were mean-pooled into a single protein-level vector. Two types of strain-level representations were derived: (i) a strain-aggregated vector obtained by averaging all protein embeddings and (ii) protein-level token embeddings that preserve individual protein granularity as sequential input to the cross-attention module, analogous to the atom-level tokens from MolE.

### Cross-attention for combined representations

Drug and bacterial token embeddings reside in fundamentally different embedding spaces. To bridge these heterogeneous modalities, we employed a bidirectional cross-attention module [32–34], in which each representation selectively attends to the most relevant features of the other. Drug tokens serve as queries to attend over bacterial protein tokens, and bacterial protein tokens serve as queries to attend over drug tokens, with residual connections applied to preserve modality-specific information. The mathematical formulation and detailed description are provided in the Supplementary *Methods, Cross-Attention for Combined Representation* section.

Beyond predictive performance, this architecture provides interpretability by identifying which bacterial proteins attend to specific drug substructures and which atomic regions of a drug are most strongly attended to by bacterial proteins. The final combined representation is constructed by concatenating (i) the token mean-pooled cross-attention outputs from both directions, (ii) the projected KEGG pathway score vector encoding strain-level metabolic capacity, and (iii) the token mean-pooled drug embedding, which maintains a balanced contribution from both modalities (Fig. 1).

**Figure 1:**
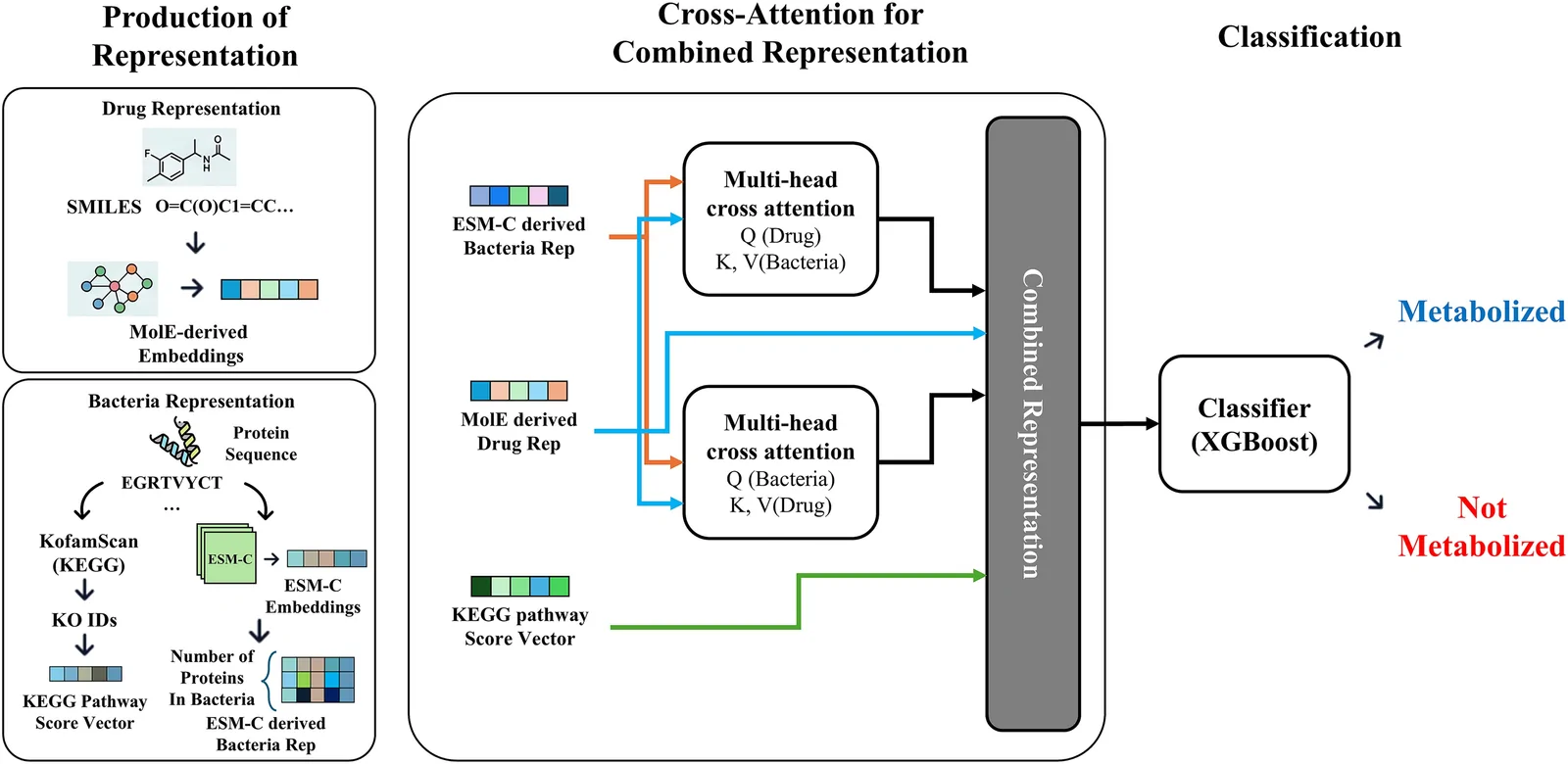
Architecture of the proposed multimodal bidirectional multi-head attention model for bacteria-specific drug metabolism prediction. The workflow consists of three stages. First, drug and bacterial representations are generated from chemical and protein data. Drug representations include simplified molecular-input line-entry system strings, physicochemical descriptors, extended-connectivity fingerprints, and atom-level molecular embeddings from MolE, a pretrained graph isomorphism network model. Bacterial representations include protein-level embeddings from ESM-C, a pretrained protein language model, and a Kyoto Encyclopedia of Genes and Genomes pathway score vector representing strain-level metabolic capacity. Second, bidirectional multi-head cross-attention models interactions between drug atom tokens and bacterial protein tokens to produce a combined representation. Third, the combined representation is classified with Extreme Gradient Boosting to predict whether the drug is metabolized or not metabolized by the bacterial strain. Some icons in this figure were sourced from Flaticon.com. Abbreviations: Bi-MHA, bidirectional multi-head attention; SMILES, simplified molecular-input line-entry system; ECFP, extended-connectivity fingerprint; KEGG, Kyoto Encyclopedia of Genes and Genomes; XGBoost, Extreme Gradient Boosting.

### Classification

The combined representation from the cross-attention modules is passed through a two-stage classification pipeline consisting of an MLP-based classifier and an XGBoost-based classifier, each serving a distinct role. The MLP-based classifier consists of multiple fully connected layers with Gaussian error linear unit (GELU) activation functions and dropout regularization. During training, its primary role is to propagate prediction loss through the network via backpropagation, thereby updating the weights of the cross-attention layers. Once the cross-attention module has been optimized through MLP training, the refined combined representations are extracted and fed into an XGBoost-based classifier. XGBoost, a gradient-boosted tree ensemble, is well-suited for capturing complex nonlinear decision boundaries.

### Data preparation and labeling

The main dataset used to train the model was derived from the study by Zimmermann et al. [35], in which 76 gut bacterial strains, representing the major phyla of the human gut microbiome, were individually incubated with 271 orally administered drugs under anaerobic conditions for 12 h, yielding 20 596 drug–bacteria interactions. To generate bacterial representations, protein sequence data for these gut bacterial strains were curated from National Center for Biotechnology Information (NCBI) Datasets, UniProt, and various literature sources. Five gut bacterial strains isolated from human feces were excluded because of the absence of strain-level information, resulting in 19 241 drug–bacteria interactions. To generate molecular representations, SMILES strings for the drugs were obtained from the main dataset and used for molecular feature extraction.

Binary labels for each drug–bacteria pair were assigned based on the drug-adaptive fold-change (FC) thresholds defined by Zimmermann et al. [35]. A pair was labeled positive if the consumed fraction exceeded its drug-specific FC threshold and negative otherwise. In addition to FC-based labeling, each drug–bacteria interaction was characterized by its false discovery rate (FDR)-corrected *P*-value (q-value), as defined in the original study [35], which assessed the statistical significance of the observed drug depletion by comparing drug intensity in drug-containing pools with that in non-drug vehicle controls. To maximize data utilization while accounting for differences in label certainty, we examined the relationship between FC-based labels and q-value significance, yielding four interpretive categories: (i) interactions with q-value *≤* 0.05 and a consumed fraction above the FC threshold, indicating that the drug was significantly metabolized by gut bacteria; (ii) interactions with q-value *≤* 0.05 and a consumed fraction below the FC threshold, indicating that no biologically meaningful metabolism occurred, as statistically confirmed against controls; (iii) interactions with q-value > 0.05 and a consumed fraction above the FC threshold, indicating that the fold change exceeded the threshold but that the observed consumption could not be statistically distinguished from that observed in non-drug controls, leaving the bacterial contribution uncertain; and (iv) interactions with q-value > 0.05 and a consumed fraction below the FC threshold, indicating that no significant drug depletion was observed.

These four interpretive categories were consolidated into two reliability categories: high-reliability data points (q-value *≤* 0.05), in which the assigned label, positive or negative, is grounded in statistically significant bacterial activity confirmed against non-drug controls; and low-reliability data points (q-value *>* 0.05), in which the label lacks such statistical confirmation. This stratification motivated the use of a reliability-aware, *λ*-scaled loss function, detailed in the *Sampling and Loss Function* section, in which each data point’s loss contribution is scaled by a factor *λ* according to its reliability category.

Because low-reliability data points lack statistically confirmed ground truth, evaluating against them would undermine the validity of the performance estimates. Accordingly, the validation and test sets were drawn exclusively from high-reliability data points, whereas low-reliability data points were used only for training. This ensured that all reported metrics reflected the model’s ability to predict interactions verified by rigorous statistical evidence.

### Sampling and loss function

To address class imbalance and the coexistence of data points with different reliability levels, we designed multiple sampling strategies and loss formulations. For deep learning models, six strategies were investigated by varying the inclusion of low-reliability data points, the application of *λ*-scaled binary cross-entropy (BCE) loss, and the use of balanced batch sampling. For traditional machine learning models, three analogous strategies were applied using class weights and sample weights. Full descriptions of all strategies are provided in the Supplementary Methods, *Sampling and Loss Function* section.

### Nested cross-validation

To evaluate model generalizability under three unseen tasks, unseen drug, unseen bacteria, and unseen interaction, we employed a nested cross-validation framework. Nested cross-validation consists of an inner loop and an outer loop, both using k-fold splitting. Before entering the loops, the main dataset was divided into three nonoverlapping partitions: an inner dataset, a validation dataset, and a test dataset. The partitioning strategy was designed to ensure strict separation according to the unseen task. In the unseen drug task, drugs assigned to the inner dataset do not appear in the validation or test datasets, and vice versa; all data points associated with a given drug are contained exclusively within a single partition. Likewise, in the unseen bacteria task, bacterial strains are assigned exclusively to one partition, ensuring that no strain appears across multiple partitions. In the unseen interaction task, drug–bacteria pairs are partitioned such that no pair present in the inner dataset appears in the validation or test datasets. This strict partitioning ensures that the model is evaluated on entirely novel entities or interactions at each stage, preventing data leakage across partitions.

In the inner loop, hyperparameters were optimized using the Optuna Python library [36] with k-fold cross-validation on the inner dataset. In the outer loop, the model was trained on the inner dataset using the optimized hyperparameters obtained from the inner loop, with early stopping monitored on the validation dataset, and was subsequently evaluated on the held-out test dataset. To reduce computational cost, hyperparameter optimization was performed only in the first outer fold, and the resulting optimized hyperparameters were fixed and reused across all remaining outer folds. Performance for each of the three metrics was reported as the mean and standard deviation across all outer folds.

### Performance metrics

To select a model with robust generalizable performance, we evaluated three metrics across the three unseen tasks. For the binary classification task, the Matthews correlation coefficient (MCC) was used because it considers all four components of the confusion matrix, true positives ($\mathrm{TP})$, true negatives $\left(\mathrm{TN}\right)$, false positives $\left(\mathrm{FP}\right)$, and false negatives $\left(\mathrm{FN}\right)$, and has been shown to provide a reliable and informative assessment, particularly for imbalanced datasets [37, 38]:


$$\mathrm{MCC}=\frac{\mathrm{TP}\times \mathrm{TN}-\mathrm{FP}\times \mathrm{FN}}{\sqrt{\left(\mathrm{TP}+\mathrm{FP}\right)\left(\mathrm{TP}+\mathrm{FN}\right)\left(\mathrm{TN}+\mathrm{FP}\right)\left(\mathrm{TN}+\mathrm{FN}\right)}}$$

Receiver operating characteristic area under the curve (ROC AUC) was also used because it evaluates a model’s overall predictive performance regardless of the selected decision threshold [39]:


$$\mathrm{ROC}\ \mathrm{AUC}={\int}_0^1\mathrm{TPR}\left(\mathrm{FPR}\right)\mathrm{dFPR}$$

where $\mathrm{TPR}$ denotes the true positive rate and $\mathrm{FPR}$ denotes the false positive rate, defined as follows:


$$\mathrm{TPR}=\frac{\mathrm{TP}}{\mathrm{TP}+\mathrm{FN}},\kern0.5em \mathrm{FPR}=\frac{\mathrm{FP}}{\mathrm{FP}+\mathrm{TN}}$$

Balanced accuracy (BAL ACC) was also used to evaluate classification performance. It is defined as the average of sensitivity, or the TP rate, and specificity, or the TN rate. BAL ACC assigns equal weight to each class regardless of prevalence, thereby providing an unbiased measure of a classifier’s ability to correctly identify both positive and negative instances in imbalanced settings [40]:


$$\mathrm{BAL}\ \mathrm{ACC}=\frac{\mathrm{Sensitivity}\left(\mathrm{TPR}\right)+\mathrm{Specificity}\left(\mathrm{TNR}\right)}{2}$$

where $\mathrm{TNR}$ denotes the true negative rate, defined as follows:


$$\mathrm{TNR}=\frac{\mathrm{TN}}{\mathrm{TN}+\mathrm{FP}}$$

To compare model architectures across multiple tasks and metrics simultaneously, a Rank Sum Metric was computed by ranking each model across all nine performance scores (MCC, ROC AUC, and BAL ACC for each of the three unseen tasks) and averaging the ranks; a lower value indicates more consistently top-ranking performance across all conditions.

## Results and discussion

### Data exploration

The main dataset used to develop our predictive model comprised 19 241 drug–bacteria interactions derived from 271 orally administered drugs and 71 gut bacterial strains after the exclusion of five strains that lacked strain-level information (Table S1). Based on the reliability stratification described previously, as detailed in the *Data Preparation and Labeling* section, each interaction was categorized as either high-reliability or low-reliability according to its FDR-corrected *P*-value. Of these interactions, 3473 were classified as high-reliability and the remaining 15 768 as low-reliability (Fig. 2A). High-reliability data points were predominantly positive-labeled (68%), whereas low-reliability data points were overwhelmingly negative-labeled (94.6%) (Fig. 2A). This reversal in class distribution across reliability categories motivated the design of multiple training strategies, as detailed in the *Sampling and Loss Function* section, that systematically varied how high- and low-reliability data points were weighted during model training. Incorporating low-reliability data was intended to maximize data utilization and enhance model learning capacity, given that excluding these data points would provide insufficient information about the negative class.

**Figure 2:**
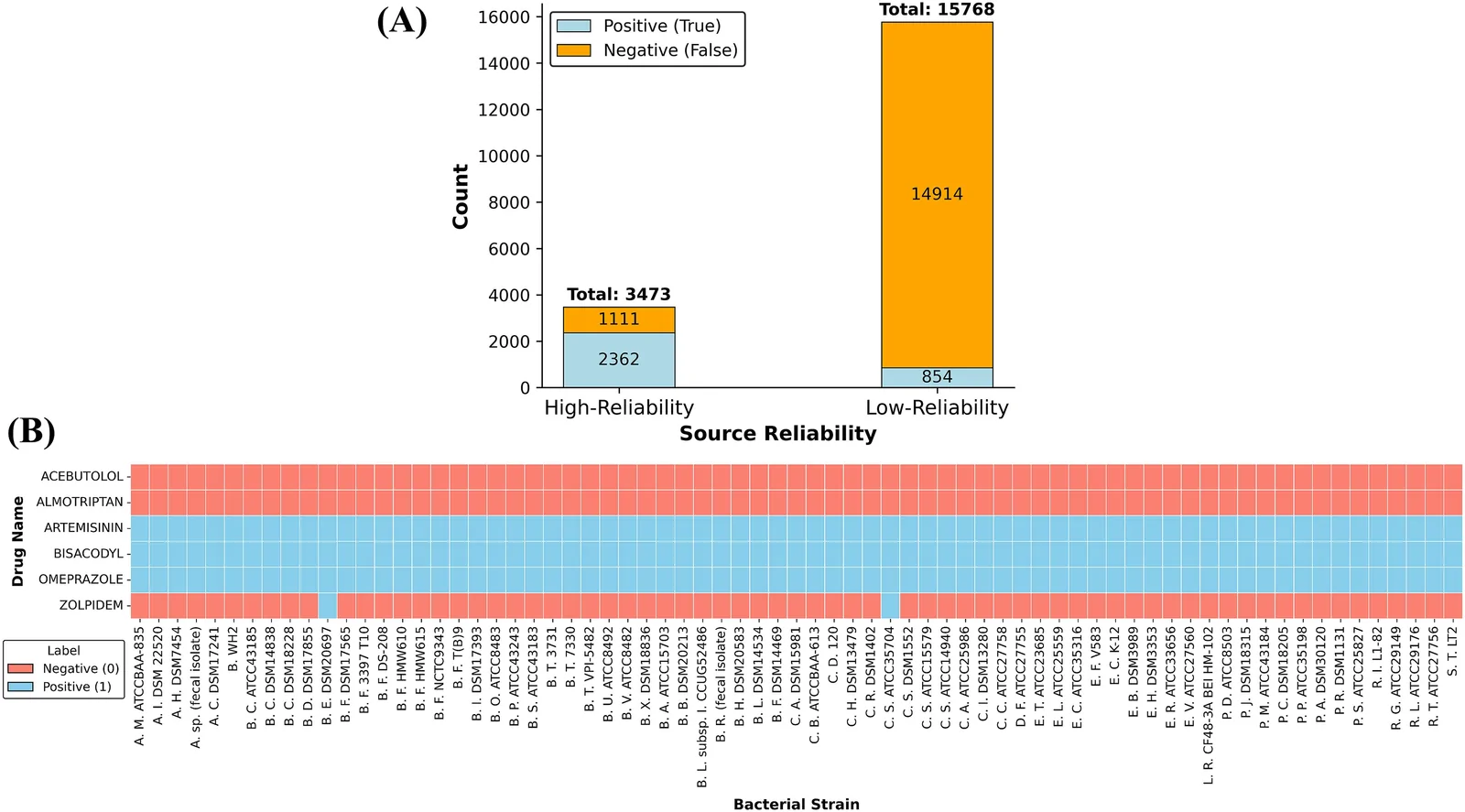
Data exploration of the main drug–bacteria interaction dataset. (A) Distribution of high-reliability and low-reliability data points, stratified by false discovery rate-corrected *P*-value and grouped by positive and negative metabolism labels. Positive labels indicate drug–bacteria pairs in which the consumed fraction exceeded the drug-specific fold-change threshold; negative labels indicate pairs below this threshold. (B) Heatmap of representative bacteria-specific drug metabolism patterns across 71 bacterial strains, showing drugs metabolized by all strains, drugs not metabolized by any strain, and drugs metabolized only by selected strains. Abbreviations: FDR, false discovery rate; FC, fold change.

To examine how metabolic activity varies across drug–bacteria interactions, we constructed a class-label heatmap based on the main dataset, which visualizes whether each of the 271 drugs was metabolized (positive) or not metabolized (negative) by each of the 71 bacterial strains (Fig. S1). This heatmap provides an overview of drug susceptibility to bacterial metabolism and strain-level metabolic activity toward specific drugs. Seven drugs, including artemisinin, bisacodyl, melphalan, and omeprazole, were metabolized by all 71 bacterial strains, whereas 66 drugs, including acebutolol, almotriptan, and amisulpride, were not depleted by any strain. However, 198 drugs exhibited strain-specific metabolism; e.g. zolpidem was not depleted by most bacterial strains but was metabolized by *Bacteroides eggerthii* and *Clostridium scindens* (Fig. 2B). This observation indicates that bacteria-specific drug metabolism occurs across diverse drug–strain interactions, supporting the rationale for developing a predictive model that resolves metabolic activity at the individual bacterial species level rather than at the community level.

### Model evaluation

Given the large number of experimental configurations generated by combinations of molecular representations, bacterial representations, and sampling and loss function strategies, we aimed to identify the model architecture with the most consistent generalization performance. To this end, we compared all model types, including traditional machine learning models (XGB and LR) and deep learning models (MLP and cross-attention variants), by averaging performance scores across all configurations within each model type and evaluating them across three unseen tasks (Table 1). Detailed per-configuration results are provided in Table S2.

**Table 1:** Model performance comparison across three unseen prediction tasks.

Model	Rank Sum Metric	MCC_Drug_	ROC AUC_Drug_	BAL ACC_Drug_	MCC_Bacteria_	ROC AUC_Bacteria_	BAL ACC_Bacteria_	MCC_Interaction_	ROC AUC_Interaction_	BAL ACC_Interaction_
Multi modal Bi-MHA	**1.78**	0.258 ± 0.023	0.690 ± 0.016	0.638 ± 0.012	**0.675 ± 0.025**	0.920 ± 0.005	**0.839 ± 0.017**	0.740 ± 0.027	0.949 ± 0.010	**0.869 ± 0.014**
Multi modal Single-MHA	2.17	**0.270 ± 0.013**	**0.703 ± 0.013**	**0.644 ± 0.007**	0.671 ± 0.025	0.917 ± 0.008	**0.839 ± 0.014**	0.734 ± 0.026	0.946 ± 0.008	0.868 ± 0.012
XGB	2.83	0.240 ± 0.025	0.675 ± 0.014	0.626 ± 0.013	0.670 ± 0.026	**0.922 ± 0.007**	0.836 ± 0.019	**0.744 ± 0.022**	**0.953 ± 0.005**	**0.869 ± 0.015**
Bi-MHA	4.56	0.204 ± 0.049	0.656 ± 0.021	0.607 ± 0.026	0.666 ± 0.025	0.916 ± 0.007	0.835 ± 0.016	0.733 ± 0.034	0.947 ± 0.012	0.867 ± 0.017
Single-MHA	4.56	0.241 ± 0.028	0.688 ± 0.023	0.628 ± 0.015	0.661 ± 0.026	0.912 ± 0.005	0.834 ± 0.017	0.727 ± 0.020	0.945 ± 0.006	0.863 ± 0.010
MLP	5.11	0.252 ± 0.073	0.680 ± 0.054	0.634 ± 0.040	0.636 ± 0.032	0.904 ± 0.012	0.819 ± 0.018	0.713 ± 0.026	0.939 ± 0.009	0.855 ± 0.013
LR	7.00	0.189 ± 0.059	0.637 ± 0.044	0.600 ± 0.032	0.563 ± 0.094	0.873 ± 0.049	0.787 ± 0.046	0.688 ± 0.040	0.932 ± 0.015	0.850 ± 0.017

Mean ± standard deviation values are reported for Matthews correlation coefficient (MCC), receiver operating characteristic area under the curve (ROC AUC), and balanced accuracy (BAL ACC) across the unseen drug, unseen bacteria, and unseen interaction tasks. The rank-sum metric was calculated by ranking each model across the nine performance scores and averaging the ranks; lower values indicate more consistent top-ranked performance. The multimodal Bi-MHA model showed the most consistent overall performance across the three tasks.

Note. Bi-MHA, bidirectional multi-head attention; LR, logistic regression; MHA, multi-head attention; MLP, multilayer perceptron; XGB, XGBoost. Bold values indicate the highest-ranked performance and underlined values indicate the second-highest-ranked performance for each metric.

As shown in Table 1, the multimodal cross-attention variants, which incorporate the KEGG pathway score vector as an additional modality, demonstrated higher overall performance consistency across the evaluation tasks, although XGB achieved highly competitive scores in specific metrics. Among the traditional machine learning models, XGB achieved competitive performance in the unseen bacteria and unseen interaction tasks (MCC_Bacteria_: 0.670 ± 0.026; ROC AUC_Bacteria_: 0.922 ± 0.007; MCC_Interaction_: 0.744 ± 0.022; ROC AUC_Interaction_: 0.953 ± 0.005), yet showed lower performance in the unseen drug task than the multimodal variants (MCC_Drug_: 0.240 ± 0.025; ROC AUC_Drug_: 0.675 ± 0.014; BAL ACC_Drug_: 0.626 ± 0.013). Logistic regression showed the lowest overall performance across all tasks and metrics.

Among the deep learning models, the cross-attention variants consistently outperformed the MLP across all three metrics in the unseen bacteria and unseen interaction tasks, whereas in the unseen drug task only the multimodal variants exceeded the MLP. Notably, multimodal variants generally outperformed their non-multimodal counterparts, indicating that encoding strain-level metabolic capacity provides complementary information to the cross-attention representations (Table 1). Within the cross-attention architecture, the multimodal Bi-MHA model achieved consistently high performance across all three unseen tasks and all three metrics. It recorded the highest MCC among the cross-attention variants in both the unseen bacteria task (MCC_Bacteria_: 0.675 ± 0.025; ROC AUC_Bacteria_: 0.920 ± 0.005; BAL ACC_Bacteria_: 0.839 ± 0.017) and the unseen interaction task (MCC_Interaction_: 0.740 ± 0.027; ROC AUC_Interaction_: 0.949 ± 0.010; BAL ACC_Interaction_: 0.869 ± 0.014). Although multimodal Single-MHA achieved marginally higher scores in the unseen drug task (MCC_Drug_: 0.270 ± 0.013; ROC AUC_Drug_: 0.703 ± 0.013; BAL ACC_Drug_: 0.644 ± 0.007), multimodal Bi-MHA recorded the second-highest performance across all three drug-task metrics (MCC_Drug_: 0.258 ± 0.023; ROC AUC_Drug_: 0.690 ± 0.016; BAL ACC_Drug_: 0.638 ± 0.012). Taken together, when evaluated across the full set of nine performance scores, multimodal Bi-MHA was the only model that consistently ranked among the top-performing models across all conditions and was therefore selected as the proposed model for subsequent analyses.

To investigate the unseen-drug performance, we stratified the fold-wise unseen-drug test predictions by the similarity between each test drug and the training drugs within that fold, using Tanimoto similarity, Bemis-Murcko scaffold novelty, MolE embedding cosine similarity, and WHO Anatomical Therapeutic Chemical (ATC) class [41–44] (Supplementary Methods, *Stratification of Unseen-Drug Test*; Supplementary Table S3A and B). Averaged across all six training strategies, in-domain drugs (Tanimoto similarity *≥* 0.3, a similar scaffold or high cosine similarity of MolE embedding) achieved higher ROC AUC than out-of-domain drugs (0.771 versus 0.668 by Tanimoto similarity; 0.736 versus 0.684 by scaffold novelty; 0.725 versus 0.637 by MolE embedding cosine similarity), and out-of-domain drugs made up 48%–78% of the unseen-drug test set. Stratification by ATC class showed a comparable range, from 0.641 for the nervous system class (52 drugs) to 0.824 for the musculo-skeletal class (13 drugs), indicating that the model performs adequately within specific applicability domains. These results indicate that the comparatively modest unseen-drug performance largely reflects the high proportion of structurally distant, out-of-domain compounds in the unseen-drug test set rather than a general inability of the model to extrapolate to novel chemical structures.

### Ablation study

To assess the impact of input representations on model performance, results were summarized by molecular and bacterial representation type and averaged across model architectures and training strategies (Table S4A and B). Across molecular representations, MolE embeddings achieved the highest MCC in the unseen drug task (0.279 ± 0.102), outperforming both physicochemical descriptors (0.265 ± 0.092) and ECFP4 fingerprints (0.165 ± 0.102), while maintaining comparable performance across the remaining tasks (Table S4A). Beyond its competitive performance, MolE produces atom-level token embeddings that enable atom-level attention analysis within the cross-attention architecture, as detailed in the *Case Study* section, offering molecular interpretability that fixed-length representations cannot provide. Across bacterial representations, one-hot encoding achieved the highest MCC in the unseen drug task (0.250 ± 0.138); however, it is inherently inapplicable to unseen bacteria and unseen interaction tasks because it cannot represent bacterial species absent from the training set (Table S4B). Among the generalizable representations, ESM mean-pooled embeddings demonstrated performance comparable to that of conventional descriptors such as CTD and KEGG pathway scores across all three tasks, while consistently outperforming CTD (e.g. unseen bacteria MCC: 0.625 versus 0.617; unseen interaction MCC: 0.715 versus 0.710). Furthermore, as a sequence-level representation, ESM embeddings offer the additional advantage of enabling token-level attention analysis within the cross-attention architecture, providing protein-level interpretability that fixed-length descriptors cannot offer. Accordingly, MolE and ESM-C embeddings were selected as the molecular and bacterial representations for the proposed model, respectively, because they provide both competitive predictive performance and token-level interpretability through the cross-attention architecture.

We also investigated how training configurations influence model performance. Six sampling and loss function strategies, detailed in the Supplementary Methods, *Sampling and Loss Function* section, were evaluated across all three unseen tasks (Fig. S2). Overall, the model showed robust performance across different training strategies. Notably, strategies using the full dataset, including both high- and low-reliability data points, did not degrade performance relative to high-reliability-only strategies. Instead, they tended to yield comparable or higher scores across most conditions, suggesting that incorporating low-reliability data with appropriate loss weighting provides additional informative signal. Based on these observations, *λ*-scaled loss with full-data training was adopted as the default configuration.

### External validation

To assess the model’s generalizability under a genuinely out-of-distribution (OOD) scenario, an external validation dataset containing only drugs and bacterial strains that were completely absent from the main training data was curated [45–48] (Supplementary Table S5A). Predictions were generated by ensembling the five models trained during the cross-validation phase.

To characterize the relationship between the main and external dataset, we examined the distributional structure of drug-bacteria pairs at the pair level. Principal component analysis (PCA) [49, 50] of standardized (z-scored) pair-level representations showed that external validation pairs were broadly distributed throughout the main dataset’s representation space (Fig. 3A); the corresponding drug- and bacterial strain-level representations are shown in Supplementary Fig. S3A and B, respectively. Specifically, the average Euclidean distance of external drug-bacteria pairs significantly exceeded the 74th percentile of the main distribution, with 81.2% (69 of 85 pairs) exceeding the 90th percentile (Fig. 3B), establishing this as a highly challenging prediction task. Detailed statistical procedures, including the standardization protocol, are provided in the Supplementary Methods, *External Validation* section.

**Figure 3:**
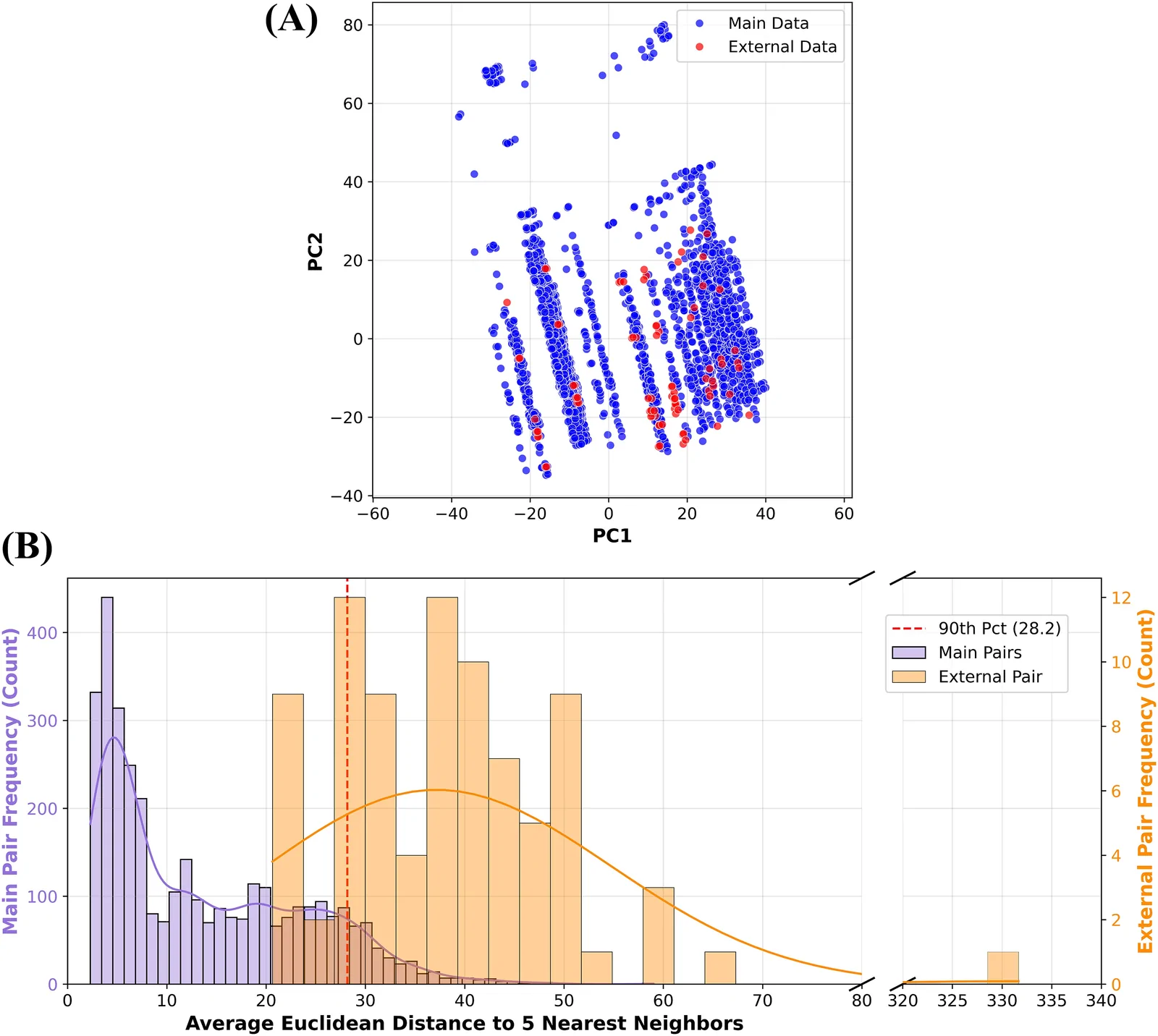
Distributional distance between the main training dataset and the external validation dataset. (A) Principal component analysis of pair-level (drug and bacteria) representations, showing that external validation drug-bacteria pairs are broadly dispersed throughout the main dataset distribution. (B) Distribution of average nearest-neighbor Euclidean distances from each drug-bacteria pair to its five nearest neighbors in the main dataset, showing that 81.2% of the external validation drug-bacteria pairs exceed the 90th percentile of the main-dataset distance distribution. Abbreviations: PCA, principal component analysis; PC, principal component.

Despite these challenges, the proposed multimodal Bi-MHA model achieved an accuracy (ACC) of 0.600 (51 of 85 pairs correctly predicted) on this external dataset (Table 2). For comparison, we evaluated a k-nearest neighbors (KNN) baseline, which was trained on the previously standardized pair-level representations of high-reliability main dataset. Given the limited size and class imbalance of the external dataset, performance was reported together with 95% confidence intervals (CIs) rather than as point estimates alone (detailed in Supplementary Methods, *External Validation* section). The proposed model achieved a ROC AUC of 0.567 (95% CI, 0.436–0.695) and an ACC of 0.600 (95% CI, 0.488–0.705), compared with a ROC AUC of 0.486 (95% CI, 0.359–0.606) and an ACC of 0.447 (95% CI, 0.339–0.559) for the KNN baseline (Table 2). Although the overlapping 95% CIs reflect statistical uncertainty attributable to the constrained sample size and the rigorous OOD setting, the proposed model consistently yielded higher point estimates across all four metrics. Moreover, stratified by bacterial strain, the proposed model generalized more consistently across the 20 unseen strains than the KNN baseline (averaged ACC by strain, 0.597 versus 0.518; Supplementary Table S5B and C). Taken together, while these results indicate an inherent performance ceiling when predicting entirely novel drug-bacteria pairs, they also show that the proposed model captures more generalizable interaction representations than a simple representation-based baseline. As the external cohort expands in future studies, this generalization advantage is expected to be characterized with greater statistical confidence.

**Table 2:** Performance of the proposed multimodal Bi-MHA model and a k-nearest neighbors (KNN) baseline on 85 out-of-distribution drug-bacteria pairs.

Model	ROC AUC [95% CI]	PR AUC [95% CI]	ACC [95% CI]	BAL ACC [95% CI]
KNN	0.486 [0.359–0.606]	0.381 [0.267–0.535]	0.447 [0.339–0.559]	0.507 [0.359–0.644]
Proposed Model (Bi-MHA)	**0.567** [0.436–0.695]	**0.489** [0.346–0.674]	**0.600** [0.488–0.705]	**0.562** [0.407–0.713]

The table shows the performance comparison for four metrics, receiver operating characteristic area under the curve (ROC AUC), precision-recall area under the curve (PR AUC), accuracy (ACC), balanced accuracy (BAL ACC). ROC AUC and PR AUC are reported with 95% confidence intervals (CI) estimated by non-parametric bootstrap (1000 resamples); ACC and BAL ACC are reported with 95% CI computed by the Clopper-Pearson exact binomial method. The proposed model showed higher point estimates than the KNN baseline across all four metrics.

Note. KNN, K-nearest neighbors; Multimodal Bi-MHA, multimodal bidirectional multi-head attention. The external validation dataset was curated from previously reported drug–bacteria metabolism data.

### Case study

To examine whether the attention weights of the multimodal Bi-MHA model align with known bacteria-specific metabolic mechanisms, we analyzed three drugs with documented bacteria-specific metabolism: digoxin, diltiazem, and sulfasalazine [1–4, 8, 35, 51].

Before qualitatively analyzing the three cases, we quantitatively evaluated the extent to which protein and atom attention signals capture meaningful patterns rather than random noise. For protein-level attention, in which drug atom tokens serve as queries to attend over bacterial proteins, we used rank-biased overlap (RBO) to evaluate whether attention-based protein rankings exhibited greater consistency across independently trained fold-specific models than expected by chance. The rankings of the top 5% and 10% of proteins showed substantially higher reproducibility than the random null baseline (top 5%: mean RBO 0.399 versus 0.073; top 10%: mean RBO 0.280 versus 0.023; Fig. S4 and B). These results suggest that the models consistently prioritize proteins across folds, supporting the interpretation that this prioritized tier reflects a reproducible, non-random signal. Similarly, for atom-level attention, in which bacterial protein tokens serve as queries to attend over drug atoms, we performed a permutation-based attention perturbation test to assess whether the learned allocation of attention to atoms contributes meaningfully to prediction. Randomly shuffling the atom-level attention weights after softmax significantly reduced predictive performance (ROC AUC: 0.715–0.687; MCC: 0.286–0.235; BAL ACC: 0.651–0.615; *P* < .001; Supplementary Table S6B). These results indicate that the learned atom-level attention weights contribute to predicting whether a drug is metabolized. Together, the protein-level consistency analysis and atom-level perturbation test support the validity of the detailed qualitative analysis of candidate proteins and molecular reaction sites (detailed methodology in Supplementary Methods, *Case Study* section).

Following these quantitative assessments, we examined the protein-level attention rankings of experimentally established drug-metabolizing proteins in the three cases: BT_4096 (WP_008760980.1) for diltiazem, Cgr2 (WP_015761237.1) for digoxin, and AzoA (WP_002-367152.1) for sulfasalazine. These proteins ranked within the 33rd–65th percentile range. These intermediate rankings were observed in the absence of explicit supervision identifying the metabolic target proteins and in the context of relatively flat softmax attention distributions across hundreds of proteins (Supplementary Table S6A). We therefore investigated whether the highly ranked proteins captured broader biological or catalytic functions relevant to drug metabolism. Guided by the cross-fold ranking consistency established by the RBO analysis, we performed Enzyme Commission (EC) number-based enrichment analysis of the top-ranked proteins (Table 3 and Supplementary Table S6C). We identified significantly enriched enzyme classes with direct or indirect functional relevance to the bacterial transformation of each drug (*P* < .05). For digoxin, oxidoreductases acting on CH-CH bonds with NAD^+^ or NADP^+^ as acceptors (EC 1.3.1) were enriched 4.93-fold in the top 10%, consistent with the reductive chemistry of its unsaturated lactone ring. For diltiazem, carboxylic ester hydrolases (EC 3.1.1) were enriched 4.94-fold among the top 10%, consistent with its metabolic deacetylation through ester hydrolysis. For sulfasalazine, thioester hydrolases (EC 3.1.2) were enriched 6.67-fold in the top 10%. This class includes an enzyme involved in menaquinone biosynthesis (EC 3.1.2.28), suggesting an indirect connection to electron-transfer processes that may support azo reduction (Table 3).

**Table 3:** EC number-based enrichment analysis of proteins ranked in the top 10% by attention weight for three case-study drug-bacteria pairs.

Drug name	Bacterial strain	EC number	Fold enrichment	*P*-Value
Digoxin	*Eggerthella Lenta* ATCC 25559	EC 1.3.1	4.93	.016
Diltiazem	*Bacteroides Thetaiotaomicron* VPI-5482	EC 3.1.1	4.94	.005
Sulfasalazine	*Enterococcus Faecalis* V583	EC 3.1.2	6.67	.028

The table presents selected enriched enzyme classes for digoxin, diltiazem, and sulfasalazine, together with the corresponding bacterial strains, fold enrichment values, and *P*-values. *P*-values were calculated using Fisher’s exact test with 2 x 2 contingency tables. The complete filtered protein set used as model input served as the background.

Note. EC, Enzyme Commission. Further details of the enrichment analysis are provided in the Supplementary Methods, *Case Study* section, and Supplementary Table S6C.

To further examine the corresponding atom-level attention patterns, we visualized fold-ensembled attention maps for top-ranked proteins belonging to these enriched EC classes, with representative maps shown in Fig. 4 and the remaining maps provided in Supplementary Fig. S5. As shown in Fig. 4, these maps highlighted reaction-relevant regions of the three drugs: the $\mathrm{\alpha}, \mathrm{\beta}$-unsaturated lactone ring of digoxin, the azo bond of sulfasalazine, and the acetyloxy group of diltiazem. For digoxin, attention peaked at the lactone carbonyl carbon (C35), adjacent to the C33-C34 double bond that undergoes enzymatic reduction (Fig. 4A). For sulfasalazine, attention peaked directly at the azo linkage (N6 and N7), the site of reductive cleavage that releases its active metabolites (Fig. 4B). For diltiazem, attention highlighted the acetyloxy group (C24 and C25), which contains the ester linkage cleaved during deacetylation (Fig. 4C).

**Figure 4:**
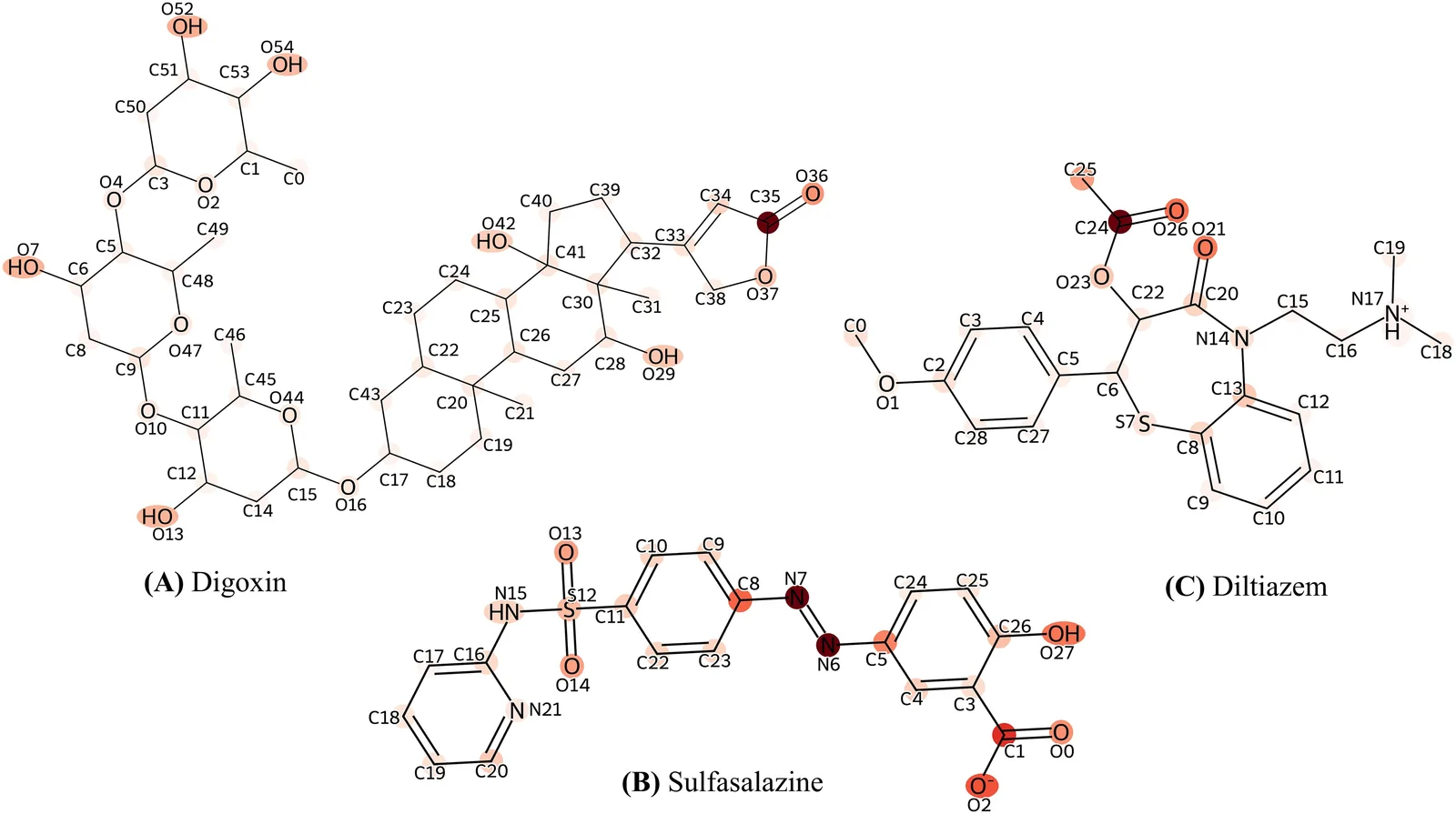
Attention-based visualization of drug–bacteria interactions for three case-study drugs. (A–C) Fold-ensembled atom-level attention maps for digoxin, sulfasalazine, and diltiazem, respectively, each shown for one representative top-ranked protein belonging to the corresponding enriched EC classes. The highlighted reaction-relevant regions are the $\mathrm{\alpha}, \mathrm{\beta}$-unsaturated lactone ring of digoxin, the azo bond of sulfasalazine, and the acetyloxy group of diltiazem.

Together, these results suggest that the model captures reproducible attention patterns with potential relevance to bacteria-specific drug metabolism, despite the absence of explicit supervision of metabolic enzymes or reaction sites. Atom-level attention highlights reaction-relevant molecular regions, with this attention channel shown to contribute measurably to prediction. Protein-level attention yields a reproducible relative ranking of candidate proteins, with the top-ranked group enriched in enzyme classes functionally related, directly or indirectly, to the corresponding drug transformations. These complementary findings support the model’s potential as a hypothesis-generation framework that could identify candidate reaction sites and prioritize biologically plausible proteins for experimental validation.

### Application

To evaluate whether the multimodal Bi-MHA model can predict MDM at the community level, we tested it on an independent dataset derived from a pilot donor’s fecal sample. This sample was cultured and screened against 438 orally administered drugs to determine whether they were metabolized (MDM+) or not metabolized (MDM−) [13]. The 436 drugs for which a canonical SMILES could be retrieved were used for prediction. A key challenge in microbiome data is incomplete taxonomic identification (Supplementary Table S7). To address this issue, we mapped the donor’s bacteria to strains in the main dataset based on shared family-level taxonomy, capturing 91.8% of the microbiome composition, as detailed in the Supplementary Methods, *Application* section. For each donor bacterium, the model calculated an initial metabolism probability by averaging the predictions of its matched family-level relatives. To reflect the complex microbiome environment, we then aggregated these individual probabilities into a single community-level prediction using a weighted average based on each bacterium’s relative abundance in the sample. The distribution of predicted probabilities showed clear separation between MDM+ and MDM− drugs (Fig. 5): MDM− drugs were concentrated at lower probabilities (median, ~0.18), whereas MDM+ drugs showed substantially higher predicted probabilities (median, ~0.44). These findings demonstrate the model’s ability to discriminate between metabolized and nonmetabolized drugs at the community level (ROC AUC: 0.749). All drug predictions with actual labels and performance metrics are shown in Table S8A. This analysis is intended as a proof-of-concept rather than a validated community-level prediction framework, as strain-level microbiome profiles were unavailable. Nevertheless, the model recovered a clear separation between metabolized and non-metabolized drugs even under this condition, and this separation was essentially unchanged across taxonomic matching levels and abundance thresholds, although performance was more sensitive to the choice of aggregation rule (Supplementary Table S8B–D). These findings indicate that a strain-level model can be aggregated into an informative community-level readout even from incompletely resolved microbiome data, and position the framework to be tested directly against strain-resolved profiles as such data become available—a necessary step toward personalized prediction of drug metabolism in clinical settings.

**Figure 5:**
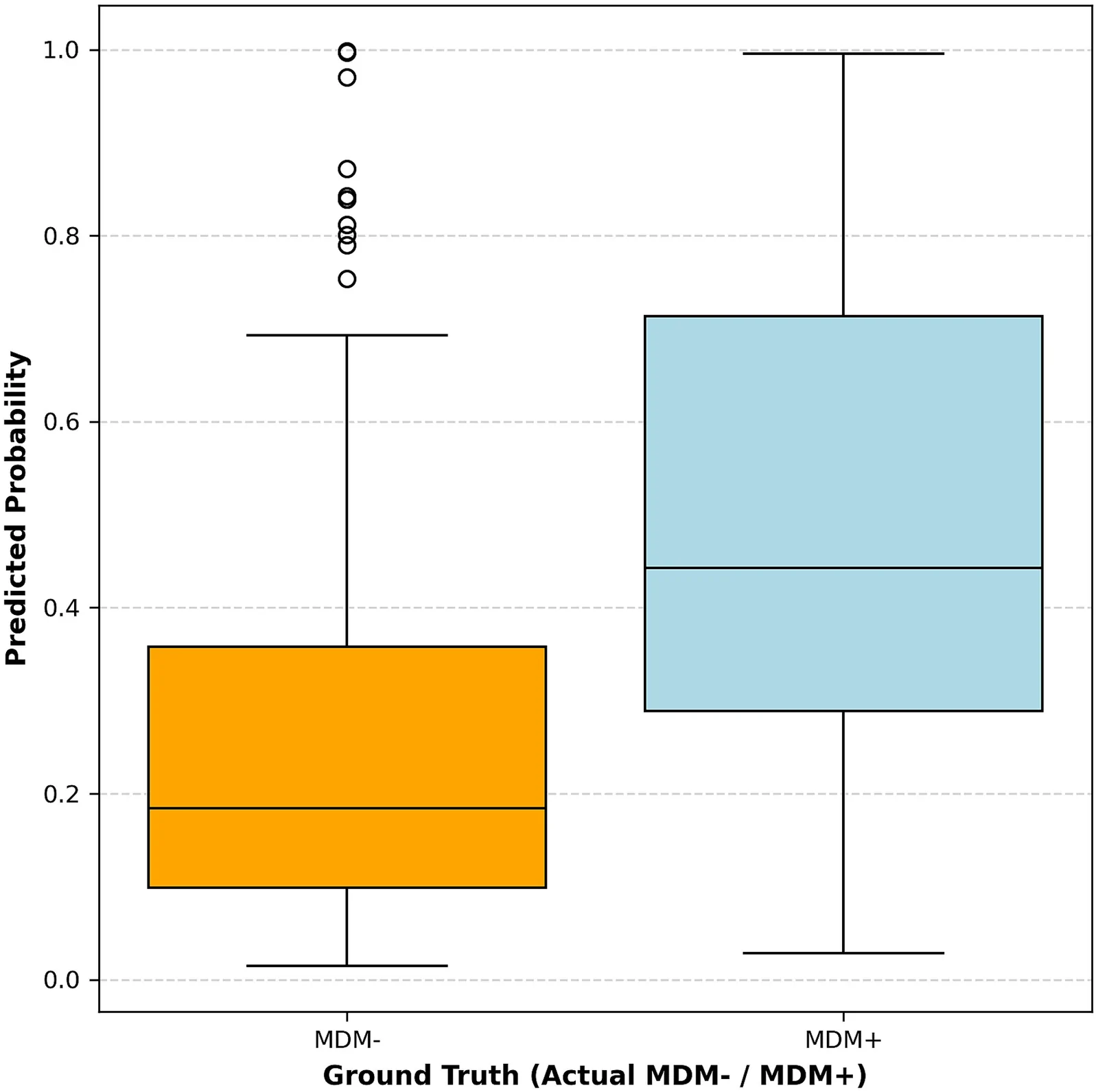
Community-level predicted probabilities for microbial drug metabolism in the independent application dataset. Box plots compare model-predicted metabolism probabilities for drugs classified as not metabolized and metabolized based on ground-truth labels from screening 436 orally administered drugs in a pilot donor’s fecal sample. Higher predicted probabilities indicate greater predicted likelihood of microbial drug metabolism at the community level. Abbreviations: MDM, microbial drug metabolism; MDM−, not metabolized; MDM+, metabolized.

## Conclusion

In this study, we developed a predictive framework for bacteria-specific drug metabolism that resolves metabolic interactions at the individual bacterial strain level. The proposed multimodal Bi-MHA model integrates pretrained molecular and protein embeddings through bidirectional cross-attention to predict bacterial drug metabolism and support interpretation at both the atom and protein levels. A reliability-aware training strategy using *λ*-scaled loss effectively leveraged both statistically confirmed and uncertain data points, enhancing robustness under data imbalance and improving generalization to OOD scenarios. The model demonstrated consistent performance across unseen drugs, bacteria, and interactions, and attention analysis of digoxin, diltiazem, and sulfasalazine highlighted reaction-relevant molecular regions and supported the prioritization of biologically plausible protein candidates. External validation on entirely novel drug–bacteria pairs indicated that this generalization extends beyond the main dataset distribution, and a community-level application illustrated how strain-level predictions can be aggregated into a microbiome-level readout. Overall, the developed framework provides interpretable metabolism maps with attention-based visualizations of metabolic sites and relative protein rankings. This tool may support drug safety assessment during early-stage development, generate mechanistic hypotheses about previously uncharacterized drug-microbe metabolic interactions, anticipate effects arising from microbial drug metabolism, and inform personalized dosing strategies that account for patient-specific microbiome compositions. Nevertheless, the current model is trained on a dataset of 71 bacterial strains and 271 drugs, and the relatively lower performance on chemically novel compounds in the unseen-drug task indicates that generalizability to broader chemical space remains a limitation to be addressed. The attention-based interpretations remain hypothesis-generating, and the catalytic roles of prioritized proteins require experimental validation. Additionally, the community application relies on family-level matching between the donor microbiome and training strains, which may obscure strain-level metabolic specificity and should be noted as a constraint on the community-level predictions. Future work should prioritize expanding the training dataset to include a more taxonomically diverse set of bacterial strains and a broader range of drug chemical scaffolds, which represents the most direct path to closing the unseen-drug performance gap. Refining the cross-attention formulation to sharpen protein-level resolution would further strengthen interpretability at the protein-level [52–54]. In line with advanced learning paradigms recently applied to related biomedical interaction tasks, large language models grounded on biomedical knowledge graphs can supply relational prior knowledge beyond the paired training data [55], offering a complementary route to the same goal, while multi-view contrastive learning for heterogeneous information fusion [56] could align the molecular and protein embedding spaces and thereby sharpen the protein-level resolution that the current formulation leaves diffuse. In parallel, experimental testing of candidate reaction sites and proteins prioritized through attention rankings and functional analysis in novel drug-bacteria pairs would further establish the framework’s utility for hypothesis generation in drug development. Additionally, improving the community-level prediction pipeline to incorporate strain-level taxonomic resolution, rather than relying on family-level matching, would enhance the specificity and reliability of personalized microbiome-based metabolism.

**Key Points**

- We present the multimodal Bi-MHA model, a bidirectional cross-attention framework that predicts drug metabolism at the individual bacterial strain level by integrating pretrained molecular and protein embeddings.
- The attention mechanism in the model provides mechanistic interpretability by identifying the candidate reaction sites and prioritizing biologically plausible protein candidates for bacteria-drug pairs.
- A reliability-aware training strategy using $\mathrm{\lambda}$-scaled loss effectively leverages both statistically confirmed and uncertain data, improving model robustness under data imbalance.
- The model generalizes to unseen bacteria and interactions and to unseen drugs within its chemical applicability domain, retaining its predictive advantage on out-of-distribution pairs, and extends, as a proof-of-concept, to community-level aggregation using microbial abundance data.

## Supplementary Material

supplementary_materials_bbag578

## Data Availability

The main dataset used in this study is provided in Supplementary Table S1, and the application dataset is provided in Supplementary Tables S7 and S8. The source code and trained model are available at https://github.com/Jaehyeon-O-Ob/Bacteria-Specific_Drug_Metabolism.
