# Supplementary material for: Prediction of bacteria-specific drug metabolism using a cross-attention-based model
Source: Brief Bioinform. 2026 Oct 19;27(5):bbag578. doi: 10.1093/bib/bbag578 (PMC13677792; doi:10.1093/bib/bbag578)
Supplement: Supplementary file 1 [file supplementary_materials_bbag578.zip › Supplementary Information.docx]

**Supplementary Information**

**Content**

**1. Supplementary Methods**

1.1. Molecular Representation

1.2. Bacteria Representation

1.3. Cross-Attention for Combined Representation

1.4. Sampling and Loss Function

1.5. Stratification of Unseen-Drug Test

1.6. External Validation

1.7. Case Study

1.8. Application

**2. Supplementary Figures**

**Supplementary Methods**

*Molecular Representation*

To comprehensively represent the chemical properties of drugs, we constructed three distinct molecular representations from SMILES strings. These representations are designed to capture drug features at different perspectives by utilizing traditional methods and pretrained deep learning model extraction.

For traditional molecular representations, we employed two approaches. First, a set of 217 physicochemical descriptors (which are E-state indices [MaxAbsEStateIndex, MaxEStateIndex, MinAbsEStateIndex, MinEStateIndex], Bulk molecular properties [qed, SPS, MolWt, HeavyAtomMolWt, ExactMolWt, NumValenceElectrons, NumRadicalElectrons], Partial charge descriptors [MaxPartialCharge, MinPartialCharge, MaxAbsPartialCharge, MinAbsPartialCharge], Morgan fingerprint density [FpDensityMorgan1, FpDensityMorgan2, FpDensityMorgan3], BCUT2D eigenvalue descriptors [BCUT2D_MWHI, BCUT2D_MWLOW, BCUT2D_CHGHI, BCUT2D_CHGLO, BCUT2D_LOGPHI, BCUT2D_LOGPLOW, BCUT2D_MRHI, BCUT2D_MRLOW], Topological and connectivity indices [AvgIpc, BalabanJ, BertzCT, Chi0, Chi0n, Chi0v, Chi1, Chi1n, Chi1v, Chi2n, Chi2v, Chi3n, Chi3v, Chi4n, Chi4v, HallKierAlpha, Ipc, Kappa1, Kappa2, Kappa3, Phi], Surface-area and MOE-type VSA descriptors [LabuteASA, TPSA, PEOE_VSA1-PEOE_VSA14, SMR_VSA1-SMR_VSA10, SlogP_VSA1-SlogP_VSA12, EState_VSA1-EState_VSA11, VSA_EState1-VSA_EState10], Constitutional, ring, and stereochemical counts [FractionCSP3, HeavyAtomCount, NHOHCount, NOCount, NumAliphaticCarbocycles, NumAliphaticHeterocycles, NumAliphaticRings, NumAmideBonds, NumAromaticCarbocycles, NumAromaticHeterocycles, NumAromaticRings, NumAtomStereoCenters, NumBridgeheadAtoms, NumHAcceptors, NumHDonors, NumHeteroatoms, NumHeterocycles, NumRotatableBonds, NumSaturatedCarbocycles, NumSaturatedHeterocycles, NumSaturatedRings, NumSpiroAtoms, NumUnspecifiedAtomStereoCenters, RingCount], Lipophilicity and molar refractivity [MolLogP, MolMR], Fragment counts [fr_Al_COO, fr_Al_OH, fr_Al_OH_noTert, fr_ArN, fr_Ar_COO, fr_Ar_N, fr_Ar_NH, fr_Ar_OH, fr_COO, fr_COO2, fr_C_O, fr_C_O_noCOO, fr_C_S, fr_HOCCN, fr_Imine, fr_NH0, fr_NH1, fr_NH2, fr_N_O, fr_Ndealkylation1, fr_Ndealkylation2, fr_Nhpyrrole, fr_SH, fr_aldehyde, fr_alkyl_carbamate, fr_alkyl_halide, fr_allylic_oxid, fr_amide, fr_amidine, fr_aniline, fr_aryl_methyl, fr_azide, fr_azo, fr_barbitur, fr_benzene, fr_benzodiazepine, fr_bicyclic, fr_diazo, fr_dihydropyridine, fr_epoxide, fr_ester, fr_ether, fr_furan, fr_guanido, fr_halogen, fr_hdrzine, fr_hdrzone, fr_imidazole, fr_imide, fr_isocyan, fr_isothiocyan, fr_ketone, fr_ketone_Topliss, fr_lactam, fr_lactone, fr_methoxy, fr_morpholine, fr_nitrile, fr_nitro, fr_nitro_arom, fr_nitro_arom_nonortho, fr_nitroso, fr_oxazole, fr_oxime, fr_para_hydroxylation, fr_phenol, fr_phenol_noOrthoHbond, fr_phos_acid, fr_phos_ester, fr_piperdine, fr_piperzine, fr_priamide, fr_prisulfonamd, fr_pyridine, fr_quatN, fr_sulfide, fr_sulfonamd, fr_sulfone, fr_term_acetylene, fr_tetrazole, fr_thiazole, fr_thiocyan, fr_thiophene, fr_unbrch_alkane, fr_urea]) was calculated to capture global molecular properties such as lipophilicity and molecular weight. Second, Extended-Connectivity Fingerprints (ECFP) with a radius of 4 and 1,024 bits were generated to encode local molecular topology and sub-structural motifs. Both representations were computed using the RDKit Python library [1].

For extracting molecular representations, MolE, a pretrained Graph Isomorphism Network (GIN)-based model, was utilized [2]. Given a molecular graph $G$ with $N$ atoms, MolE produces an atom-level embedding for each atom $i$

$$h_{i}=\text{MolE}\left( G \right)_{i}\in R^{d_{m}},\quad i=1,\ldots,N$$

where $d_{m}$ denotes the molecular representation dimension. From these atom-level embeddings, we construct two types of representations. First, a mean-pooled embedding is computed to produce a single fixed-length vector per molecule:

$$\bar{h}=\frac{1}{N}\sum_{i=1}^{N} h_{i}\in R^{d_{m}}$$

Second, each atom-level embedding is treated as an individual token, and the complete set is organized as a token embedding:

$$H=\left[ h_{1};h_{2};\ldots;h_{N} \right]\in R^{N\times d_{m}}$$

Notably, while the mean-pooled representation $\bar{h}$ compresses molecular information into a single vector of dimension $d_{m}$, the token embedding $H$ preserves the atom-level granularity as a sequence of $N$ tokens for each molecule. These two forms serve distinct roles in various models: the token embedding is used as input to the cross-attention module, while the mean-pooled vector is fed into traditional machine learning model.

*Bacteria Representation*

To represent the 71 gut bacterial strains, we constructed four distinct bacterial representation methods derived from strain names and their protein sequences.

First, each bacterial strain was represented as a one-hot encoded vector based on its taxonomic identity, serving as a baseline representation that encodes genus-strain-level categorical information without biological context.

Second, to capture the physicochemical properties of bacterial proteins, Composition, Transition, and Distribution (CTD) descriptors were computed for each protein sequence within a strain. The CTD framework, originally proposed by Dubchak et al., encodes three complementary aspects of protein sequence composition: Composition (the fraction of residues belonging to each physicochemical group), Transition (the frequency of transitions between groups along the sequence), and Distribution (the positional distribution of residues within each group). These descriptors categorize the 20 standard amino acids into three groups based on seven physicochemical properties, including hydrophobicity, polarizability, and charge [3, 4]. For each protein within a strain, a set of 147 CTD values was calculated using the protpy Python library [5]. Proteins containing non-standard amino acids or with insufficient sequence length were excluded from this calculation. The resulting protein-level CTD vectors were then aggregated via mean pooling to produce a single strain-level representation.

Third, inspired by the approach of Algavi et al. [6], a pathway-based representation was applied by using the Kyoto Encyclopedia of Genes and Genomes (KEGG) database, which integrates molecular-level information into functional representations of biological systems such as metabolic and signaling pathways [7]. For each bacterial strain, all protein sequences were assigned KEGG Orthology IDs (KO IDs) using KofamScan, a Hidden Markov Model(HMM)-based homology search tool [8]. Then 204 unique KEGG pathways relevant to bacterial organisms were identified and quantified the functional profile of each strain as a pathway-based score vector. Each element of this vector reflects the number of unique KO groups present within a specific pathway; KO IDs associated with multiple pathways were proportionally distributed among those pathways to avoid overrepresentation. This process transformed the protein-level functional annotations into a structured strain-level representation.

Fourth, to obtain context-rich protein representation, we utilized ESM-C (Evolutionary Scale Modeling-Cambrian) 600M [9], a pretrained protein language model with 600 million parameters optimized for representation learning, which has demonstrated improved performance over ESM-2 [10–12]. Prior to extracting embedding vectors, proteins within each strain were filtered to retain only those assigned KO IDs belonging to the “Metabolism” section of KEGG pathways, as proteins unrelated to metabolic functions are unlikely to contribute to drug metabolism prediction and may introduce noise. Let $M$ denote the number of selected proteins after filtering. For each selected protein $j$ with amino acid sequence $S_{j}$ of length $L_{j}$, ESM-C produces residue-level embeddings:

$$r_{j,k}=\text{ESM-C}\left( S_{j} \right)_{k}\in R^{d_{p}},\quad k=1,\ldots,L_{j}$$

where $d_{p}$ denotes the protein representation dimension. A mean-pooled embedding across all residues is computed to obtain a single vector per protein:

$$p_{j}=\frac{1}{L_{j}}\sum_{k=1}^{L_{j}} r_{j,k}\in R^{d_{p}}$$

From these protein-level embeddings, two types of strain-level representations that serve as bacteria representations are constructed. First, a strain-level aggregated vector is obtained by averaging:

$$\bar{p}=\frac{1}{M}\sum_{j=1}^{M} p_{j}\in R^{d_{p}}$$

Second, each protein embedding is treated as an individual token, and the complete set is organized as token embeddings:

$$P=\left[ p_{1};p_{2};\ldots;p_{M} \right]\in R^{M\times d_{p}}$$

Similar to the molecular token embeddings $H\in R^{N\times d_{m}}$, the protein token embeddings $P$ preserve individual protein-level granularity as a sequence of $M$ tokens within a strain (hereafter referred to as bacterial token embeddings), whereas the strain-aggregated vector $\bar{p}$ is later appended to the combined representation to retain global bacterial context.

*Cross-Attention for Combined Representations*

The following steps describe how the combined representation is constructed through our predictive model design.

First, drug and bacteria token embeddings are projected into a common latent space of dimension $d_{l}$ through a learnable linear layer to apply cross-attention between them. Because those token embeddings are derived from different model architectures, they reside in different dimensional spaces and cannot be directly combined. This projection aligns the two representations dimensionally, making them compatible for attention computation. Given the bacteria token embedding matrix $P\in R^{M\times d_{P}}$ and drug token embedding matrix $H\in R^{N\times d_{H}}$, the projections are:

$$Z_{P}=W_{P}\cdot P_{d_{P}}+b_{P}, whereZ_{P}\in R^{M \times d_{l}}$$

$$Z_{H}=W_{H}\cdot H_{d_{H}}+b_{H}, whereZ_{H}\in R^{N \times d_{l}}$$

where $W_{P}\in R^{d_{l}\times d_{P}},W_{H}\in R^{d_{l}\times d_{H}}$ are learnable weight matrices and $b_{P},b_{H}\in R^{d_{l}}$ are bias vectors.

Second, the projected drug and bacteria embeddings are fused through a bidirectional cross-attention module. In cross-attention, a sequence attends to a different sequence, while self-attention, a sequence attends to itself. Specifically, the query Q is derived from one embedding, while the key K and value V come from the other, allowing each representation to identify which parts of its counterpart are most relevant. To capture both directions of interaction, we employ two separate cross-attention modules. In detail, the cross-attention from bacteria to drug is defined as:

$$A\mathrm{ttn}\left( P\to H \right)=FFNN\left( \mathrm{softmax}\left( \frac{Q_{P}K_{H}^{T}}{\sqrt{d_{l}}} \right)V_{H} \right)$$

and the cross-attention from drug to bacteria is defined as:

$$\mathrm{Attn}\left( H\to P \right)=FFNN\left( \mathrm{softmax}\left( \frac{Q_{H}K_{P}^{T}}{\sqrt{d_{l}}} \right)V_{P} \right)$$

Furthermore, the original projected embedding is added back to the cross-attention output to prevent dilution of the original, modality-specific information – a technique known as residual connection:

$$Z_{CA_{P}}=Z_{P}+Attn\left( P\to H \right)$$

$$Z_{CA_{H}}=Z_{H}+Attn\left( H\to P \right)$$

Third, the final representation is assembled by concatenating three components: (1) the mean-pooled bacteria embedding $\bar{Z}_{CA_{P}}$ and drug embedding $\bar{Z}_{CA_{H}}$ – obtained by mean-pooling $Z_{CA_{P}}$ over protein-level tokens and $Z_{CA_{H}}$ over atom-level tokens, respectively by preceding bidirectional cross-attention module, (2) the projected KEGG pathway-based score vector $\bar{Z}_{\mathrm{KEGG}}$, and (3) the projected drug embeddings $\bar{Z}_{H}$. Because the bacteria embeddings have already been processed by the cross-attention module, appending their mean-pooled counterpart would introduce redundancy from the same information source. Instead, the KEGG pathway-based score vector is incorporated, as it encodes the strain’s functional metabolic capacity at the pathway level and provides non-redundant complementary context. This vector is projected into the shared latent space prior to concatenation:

$$\bar{Z}_{\mathrm{KEGG}}=W_{\mathrm{KEGG}}\cdot E_{\mathrm{KEGG}}+b_{\mathrm{KEGG}}$$

where $E_{\mathrm{KEGG}}\in R^{d_{KEGG}}$ is the KEGG pathway-based score vector, $W_{\mathrm{KEGG}}\in R^{d_{l}\times d_{\mathrm{KEGG}}}$ and $b_{\mathrm{KEGG}}\in R^{d_{l}}$ are learnable parameters, and $\bar{Z}_{\mathrm{KEGG}}\in R^{d_{l}}$

Since the KEGG vector is a bacteria-only representation with no direct drug counterpart in the concatenated components – whereas both cross-attention embeddings already encode mutual interaction information – the mean-pooled drug embedding $\bar{Z}_{H}$, which is obtained by averaging over atom-level projected token embeddings, is appended to ensure that drug intrinsic features are directly represented in the final vector, maintaining a balanced contribution from both modalities. The combined representation is thus constructed as:

$$\bar{Z}_{\mathrm{final}}=concat\left( \bar{Z}_{CA_{P}},\bar{Z}_{CA_{H}},\bar{Z}_{\mathrm{KEGG}},\bar{Z}_{H} \right), where \bar{Z}_{final}\in R^{4d_{l}}$$

*Classification*

The combined representation $\bar{Z}_{\mathrm{final}}$ is passed through a two-stage classification pipeline consisting of a multilayer perceptron (MLP)-based classifier and an XGBoost-based classifier, each serving a distinct role. The MLP-based classifier consists of multiple fully connected layers with GELU activation functions and dropout regularization. Its primary role during training is to propagate the prediction loss back through the network via backpropagation, thereby updating the weights of the cross-attention layers. Once the cross-attention module has been optimized through MLP training, the refined combined representations are extracted and fed into an XGBoost-based classifier. XGBoost, a gradient-boosted tree ensemble, is well-suited to capture complex nonlinear decision boundaries. The predicted drug metabolism state for a given drug-bacteria pair is thus:

$$\left( \hat{y},\hat{y}_{\mathrm{prob}} \right)=XGBoost\left( \bar{Z}_{\mathrm{final}} \right)$$

where $\hat{y}\in\{0,1\}$ denotes the predicted drug metabolism label for a given drug-bacteria pair, and $\hat{y}_{\mathrm{prob}}\in\left[ 0,1 \right]$ denotes the corresponding predicted probability from the XGBoost-based classifier.

*Sampling and Loss Function*

To address two key challenges inherent in the main dataset – class imbalance between positive and negative labels, and the coexistence of high-reliability and low-reliability data points with different label certainty – we designed multiple data sampling strategies and loss formulations applied during model training.

For the deep learning models (including cross-attention-based model and the MLP), five strategies were investigated

(A) Binary cross-entropy (BCE) loss was computed over the entire main dataset – including both high-reliability and low-reliability data points – with pos_weight parameter in BCE set inversely proportional to class frequency:

$$BCE=-ylog\left( \hat{y} \right)-\left( 1-y \right)\log\left( 1-\hat{y} \right)$$

where $y$ denotes labels and $\hat{y}$ denotes predicted values from the models.

(B) The full main dataset was used for training. The BCE loss for low-reliability data points ($\mathrm{BC}E_{low}$) was scaled by a factor $\lambda$ (where 0 < $\lambda$ < 1) before being added to the unscaled BCE loss of high-reliability data points ($\mathrm{BC}E_{\mathrm{high}}$), placing greater emphasis on the latter. In addition, balanced batch sampling was applied using a weighted random sampler to construct each training batch with an equal proportion of positive and negative labels, further addressing class imbalance at the batch level:

$$\mathrm{BC}E_{\lambda}=BCE_{\mathrm{high}}+\lambda\cdot BCE_{low}$$

(C) Identical to strategy (B), except that balanced batch sampling was removed, allowing the natural class distribution to be preserved in each training batch.

(D) Only high-reliability data points were included in training, entirely excluding low-reliability data points, with balanced batch sampling applied as in strategy (B).

(E) Identical to strategy (D), except that balanced batch sampling was removed.

(F) Identical to strategy (A) without pos_weight parameter in BCE set.

To compare with deep learning models, three analogous strategies were applied to the traditional machine learning baselines – XGBoost (XGB) and Logistic Regression (LR):

(i) All data points (both high-reliability and low-reliability) were used for training, with class weights provided to the model to account for class imbalance.

(ii) Only high-reliability data points were used, with class weights applied to balance the remaining class distribution.

(iii) All data points were included, but each instance was assigned an individual sample weight reflecting its reliability category: high-reliability data points received a sample weight of 1.0, while low-reliability data points received a reduced sample weight (< 1.0). These sample weights were supplied to the model’s sample weight parameter, analogous to the $\lambda$-scaled loss approach use in strategy (B).

*Stratification of Unseen-Drug Test*

To evaluate whether unseen-drug test performance depends on chemical similarity to the training set, the fold-wise predictions from the completed unseen-drug cross-validation were stratified using four complementary criteria, each computed independently within each of the five outer folds using the same train-test drug partitions as the main evaluation. First, Tanimoto similarity was calculated between ECFP4 fingerprints of each test drug and all training drugs in the corresponding fold; the maximum similarity per test drug was retained, and drugs with a maximum similarity $\geq$ 0.3 were classified as in-domain, with the remainder classified as out-of-domain according to the OECD QSAR guidelines [13]. Second, Murcko scaffolds were extracted for all drugs, and test drugs sharing an identical scaffold with at least one training drug in the same fold were classified as scaffold-similar, while the rest were classified as scaffold-novel with RDKit Python library (version 2025.3.1). Third, pairwise cosine similarity was computed between mean-pooled MolE embeddings of test and training drugs, standardized using parameters fit on the training set of each fold, and the maximum similarity per test drug was used to split the test set at a threshold of 0.7, corresponding approximately to the median, into high- and low-similarity groups. Fourth, World Health Organization Anatomical Therapeutic Chemical (ATC) level-1 codes were retrieved for each drug using the RxNav RxClass API (National Library of Medicine), with drug names standardized by removing common salt suffixes and mapping United States Adopted Names to International Nonproprietary Names where necessary; drugs without a retrievable code were labeled unclassified. For each stratification, ROC AUC, MCC, and balanced accuracy were computed within each subgroup and averaged across the five outer folds and six training strategies, and the full results are reported in Supplementary Tables S3A and S3B.

*External Validation*

The external validation dataset was curated from diverse published literature sources [14–17] , containing drug–bacteria interactions involving only drugs and bacterial strains completely absent from the main dataset (Supplementary Table S5A). To maintain labeling consistency with the main dataset, only studies reporting a consumption (depletion)-based readout of drug biotransformation were included, and each drug-bacteria pair was labeled as positive (metabolized) or negative (not metabolized) according to the statistical significance of metabolism reported in the corresponding source study. Molecular and bacteria representations were extracted using the same procedures described in the *Molecular Representation* and *Bacteria Representation* sections. For prediction, the five models obtained from each fold of the optimized unseen drug cross-validation were ensembled. Prior to prediction, we confirmed that none of the drugs or bacterial strains in the external validation dataset appeared in any of the five training folds, thereby guaranteeing full independence from the training data.

To quantitatively assess whether the external validation dataset constitutes a more challenging prediction task, drug and bacteria representations were standardized (z-scored) using scaling parameters fit on the high-reliability main dataset and applied to the external dataset without refitting by utilizing the scikit-learn Python library [18], and we analyzed the distributional distance between the main and external datasets using principal component analysis (PCA) and nearest-neighbor Euclidean distance statistics. PCA was applied to the standardized combined representations (drug- and bacteria representation), revealing that drug-bacteria pairs in the external validation dataset are broadly dispersed throughout the main dataset distribution. Then corresponding drug- and bacterial strain-level representations are shown separately using the same method in Supplementary Figures S3A and S3B. Basically, although external drugs were different from main drugs and their representations did not fully overlap in PCA space, their representations remained closely positioned within the surrounding representation space (Figure S3A). For bacterial strains, although some external strains shared the same genus and species as strains in the main dataset, they differed at the strain level; consequently, their representations did not fully overlap, and a noticeable distance was observed in PCA space (Figure S3B).

To quantify this separation, a nearest-neighbor analysis was conducted using the standardized combined drug-bacteria pair representation, using the same scaling parameters described above. For each drug-bacteria pair in the high-reliability main dataset, the five nearest neighbors within the main dataset were identified, excluding the pair itself, and the average Euclidean distance to these neighbors was computed. The same procedure was applied to each drug-bacteria pair in the external validation dataset, measuring the average distance to its five nearest neighbors in the main dataset. The distribution of average nearest-neighbor distances for the external validation pairs exceeded the 74th percentile of the main distribution, with 81.2% (69 of 85 pairs) exceeding the 90th percentile (Figure 3B), confirming that the external drug-bacteria pairs reside in statistically distant regions of the combined representation space.

For the k-nearest neighbors (KNN) baseline, the previously standardized pair-level combined representations of the high-reliability main dataset were used as the training set, and the corresponding standardized pair-level representations of the external validation dataset were used as the test set. The KNN model was trained on the high-reliability main dataset with the number of neighbors set to k=5 and the Euclidean distance metric. For each external validation pair, the predicted probability was calculated as the proportion of positive labels among its five nearest neighbors in the main dataset, and a probability $\geq$ 0.5 was classified as positive; these predictions were used to compute the KNN baseline performance reported in Table 2.

To provide a more cautious interpretation, 95% confidence intervals were calculated for all performance metrics. For accuracy and balanced accuracy, which are proportion-based metrics, exact binomial confidence intervals were computed using the Clopper-Pearson method[19, 20]; the confidence interval for balanced accuracy was obtained by averaging the Clopper-Pearson intervals separately calculated for sensitivity and specificity. For receiver operating characteristic area under curve (ROC AUC) and precision-recall area under curve (PR AUC), which are not binomial proportions, 95% confidence intervals were estimated using a non-parametric bootstrap procedure with 1,000 resamples. These confidence intervals are reported alongside each metric for both the proposed model and the KNN model in Table 2, allowing their performance to be compared while accounting for sampling uncertainty.

*Case Study*

Attention analyses comprised quantitative evaluation of the two attention channels followed by functional interpretation and molecular visualization. Atom-level attention was extracted from Cross 1, in which bacterial protein tokens serve as queries and drug atom tokens serve as keys and values. Protein-level attention was extracted from Cross 2, in which drug atom tokens serve as queries and bacterial protein tokens serve as keys and values. Quantitative analyses used the unseen-drug cross-validation models and the datasets specified below. Subsequent interpretation focused on digoxin–Eggerthella lenta ATCC 25559, diltiazem–Bacteroides thetaiotaomicron VPI-5482, and sulfasalazine–Enterococcus faecalis V583. No explicit supervision identifying metabolic enzymes or reaction sites was used during training.

**Permutation-based perturbation of atom-level attention**

The contribution of the learned atom-level attention allocation to prediction was evaluated using a permutation-based attention perturbation test. For each of the five unseen-drug test folds, post-softmax Cross 1 attention weights were randomly permuted along the atom axis within each drug–bacteria pair. The resulting representations were passed through the corresponding fold-specific classifier without retraining. This procedure was repeated 1,000 times per fold. For each permutation replicate, ROC AUC, Matthews correlation coefficient (MCC), and balanced accuracy were calculated and averaged across the five folds. Original fold-averaged performance was compared with the distribution obtained under repeated perturbations. Empirical permutation p-values were calculated as (b + 1)/(B + 1), where B = 1,000 and b denotes the number of permutation replicates whose fold-averaged performance equaled or exceeded the original fold-averaged performance. The evaluation is reported in Supplementary Table S6B.

**Cross-fold ranking consistency for protein-level attention.**

The reproducibility of relative protein rankings was evaluated using rank-biased overlap (RBO) for 1,000 drug–bacteria pairs sampled from the high-reliability main dataset. For each pair, the analysis used the four fold-specific models whose training sets included the corresponding drug; the fold in which that drug was held out was excluded. At each threshold (top 5% and top 10%), the ensemble attention ranking defined a common set of k proteins. These same proteins were then ordered according to each of the four fold-specific attention rankings. RBO was calculated for all six pairwise comparisons between the four rankings and averaged to obtain one score per drug–bacteria pair at each threshold. A random baseline was generated by independently sampling k proteins from the same input protein pool for each fold and randomizing their order, followed by the same pairwise RBO calculation and averaging. The distributions of model-derived and random-baseline scores were compared separately at each threshold (Supplementary Figure S4).

**KO-to-EC mapping and enrichment analysis.**

For each case-study pair, the protein pool comprised all proteins retained for model input after the metabolism-related KO filtering described in the Bacteria Representation section. Proteins were ordered by their mean fold-specific attention rank across the five models, with smaller mean ranks indicating higher priority. The top 5% and top 10% were selected, rounding the number of proteins upward to the nearest integer. These complete filtered input protein pools, rather than the unfiltered strain proteomes, served as the enrichment backgrounds.

KEGG Orthology (KO) annotations were mapped to Enzyme Commission (EC) numbers. Enrichment was evaluated at both the full four-component EC-number level and the three-component sub-subclass level. Membership was defined at the protein level: each protein was counted at most once within a given EC category, while a protein associated with multiple categories could contribute to each corresponding category. Proteins without a mapped EC number remained in the background and were counted as non-members of the tested EC category.

For each EC category and threshold, a 2 x 2 contingency table cross-classified proteins by membership in the top-ranked set and membership in the EC category. Let N denote the number of proteins in the background, k the number in the top-ranked set, M the number assigned to the EC category in the background, and ‘a’ the number assigned to that category in the top-ranked set. The four cell counts were a (top-ranked, category member), k − a (top-ranked, non-member), M − a (remaining proteins, category member), and N − k − M + a (remaining proteins, non-member). A one-sided Fisher’s exact test was used to test for overrepresentation in the top-ranked set. Fold enrichment was calculated as (a/k)/(M/N). Categories with fold enrichment greater than 1 and an unadjusted p-value < 0.05 were considered nominally enriched. Enriched categories were examined for direct or indirect functional relevance to the documented transformation of each drug. Selected categories are presented in Table 3, with additional enrichment results in Supplementary Table S6C.

**Qualitative interpretation of atom-level attention: fold-ensembled molecular maps.**

For each case-study pair, top-ranked proteins belonging to the enriched EC classes selected for functional interpretation were used to examine protein-specific atom-level attention. Cross 1 attention matrices were extracted from the five fold-specific models, with protein and atom identities aligned across folds, and averaged element-wise. The row corresponding to each selected protein was extracted from the ensemble matrix to obtain its attention profile over the drug atoms. Thus, each profile retained the atom-level attention associated with an individual protein rather than averaging across the selected proteins.

The resulting atom-level profiles were mapped onto two-dimensional drug structures using the atom indices corresponding to the model input. Atom shading represented relative attention within each map, with darker red indicating higher attention. The highlighted regions were qualitatively compared with molecular regions involved in documented bacterial transformations. One representative protein-specific map per drug is presented in Figure 4, and maps for the remaining selected proteins are provided in the supplementary Figure S5.

*Application*

The microbiome composition reported in the application dataset [34] contained bacterial strains annotated at varying taxonomic resolutions: while some strains were fully characterized from phylum to strain level, others were only identifiable to the genus or family level. In addition, each bacterial strain in the microbiome was accompanied by its relative proportion within the community.

To leverage this compositional information while addressing incomplete strain-level annotations, a taxonomic matching procedure between the bacterial strains in the application dataset and those in the main dataset was conducted. After excluding strains with insufficient taxonomic information, approximately 91.8% of the microbiome composition was found to share family-level identity with bacterial strains in the main dataset. Accordingly, each bacterial strain in the application dataset was matched to all main dataset strains that shared the same taxonomic classification up to the family level. This matching procedure yielded between 1 and 23 matched main dataset strains per application dataset strain (Supplementary Table S7).

Using the matched strains, predictions were generated through a two-step aggregation procedure. In the first step, for each of the 48 bacterial strains in the application dataset, the proposed model predicted the metabolism probability—rather than a binary label—for each drug across all matched main dataset strains, and the resulting probabilities were averaged to obtain a single predicted probability per application bacterial strain. For example, if a given application bacterial strain was matched to 10 main dataset strains, the model predicted the drug metabolism probability for all 10 strains, and the mean of these 10 predicted probabilities served as the representative probability for that application bacterial strain.

In the second step, the per-strain predicted probabilities were aggregated into a single microbiome-level prediction for each drug by computing a weighted average, where the weight for each bacterial strain corresponded to its relative proportion within the microbiome. The resulting weighted probability was used as the final prediction: drugs with a weighted probability $\geq$ 0.5 were classified as MDM+, while those with a weighted probability $<$ 0.5 were classified as MDM−. All per-drug predicted probabilities and labels are provided in Supplementary Table S8A.

Because the community-level prediction depends on several pipeline choices that cannot be resolved from the available data, we evaluated the stability of the resulting readout along three axes, holding the trained model and its per-strain predictions fixed and varying only the aggregation procedure.

First, the taxonomic level at which application strains were matched to main dataset strains was varied (Supplementary Table S8B). In addition to the family-level matching used in the main analysis, we constructed an order-level mapping and a hierarchical mapping that assigns each application strain to genus-level relatives where available and falls back to family level otherwise. Under the hierarchical scheme, 39 of 62 application strains were matched at the genus level (48.9% of total relative abundance) and 11 at the family level (43.0%), with 12 remaining unmatched (8.1%). Genus-level matching resolved a more taxonomically specific set of main dataset strains than family-level matching for 17 of the 50 matched strains; for the remainder, the two schemes resolved to identical strain sets, because several families represented in the 71-strain main dataset contain only a single genus. Order-level matching, being coarser, covered 95.8% of the total relative abundance. Second, application strains falling below relative-abundance thresholds of 1%, 5%, and 10% were excluded before aggregation, evaluated on the family-level mapping (Supplementary Table S8D). Third, four aggregation rules were compared, formed by crossing the within-match step (mean versus maximum across the matched main dataset strains of a given application strain) with the across-community step (abundance-weighted average versus maximum across application strains) (Supplementary Table S8C).

Discrimination between metabolized and non-metabolized drugs was essentially unchanged across taxonomic matching levels, with ROC AUC of 0.747, 0.749, and 0.751 for hierarchical, family-level, and order-level matching, respectively. Notably, MCC and Balanced Accuracy under hierarchical matching were identical to those obtained under family-level matching (MCC: 0.292 and Balanced Accuracy: 0.663), despite genus-level matching having refined the matched strain set for 17 application strains. Performance was likewise stable across abundance thresholds (ROC AUC: 0.748-0.749; MCC: 0.286-0.292; Balanced Accuracy: 0.649-0.663), indicating that the signal is not considerably driven by the inclusion of low-abundance taxa. For the aggregation rule, the mean-and-weighted-average rule used in the main analysis achieved the highest MCC (0.292) and the second highest ROC AUC and Balanced Accuracy (0.749 and 0.663, respectively), whereas taking the maximum at both stages reduced these to 0.093, 0.712, and 0.543.

Taken together, these results indicate that the community-level readout is largely insensitive to the taxonomic matching level and abundance threshold used, and that among the aggregation rules examined the mean-and-weighted-average procedure adopted in the main analysis is best supported under class imbalance. Notably, increasing taxonomic resolution to the genus level, which refined the matched strain set for 17 application strains, left the binary predictions unchanged. These analyses probe the robustness of the aggregation procedure; whether representing an application strain by its family-level relatives adequately reflects its true metabolic capacity cannot be assessed without strain-resolved microbiome profiles, and remains the principal issue to solve in this proof-of-concept application.

**Supplementary Figures**

Figure S1. Class label heatmap of drug-bacteria metabolic interactions. The heatmap displays the binary metabolic state (Positive – light blue: bacterial metabolized; Negative – salmon: not metabolized) for each of the 271 orally administered drugs (x-axis) across 71 gut bacterial strains (y-axis). Labels were assigned based on drug-adaptive fold-change thresholds as defined in the main dataset. Drugs are ordered to highlight the spectrum of metabolic susceptibility, ranging from universally metabolized drugs to those not depleted by any strain, with bacteria-specific metabolism patterns visible in between.

Figure S2. Ablation study of training strategies across three unseen tasks. Performance comparison (MCC, ROC AUC, BAL ACC) of six sampling and loss function strategies (Strategies A to F) evaluated on unseen drug, unseen bacteria, and unseen interaction tasks. Strategies employing the full dataset consistently outperformed high-reliability-only strategies, and $\boldsymbol{\lambda}$-scaled loss without balanced batch sampling (Strategy C) achieved the highest performance in the unseen drug task.

Figure S3. Principal component analysis (PCA) of drug and bacterial strain representations, examined separately using the same method as in Figure 3A. (A) PCA of drug representations, showing that although external validation drugs were chemically distinct from main-dataset drugs and their representations did not fully overlap, they remained closely positioned within the surrounding representation space. (B) PCA of bacterial strain representations, showing that although several external strains shared the same genus or species as strains in the main dataset, they differed at the strain level; consequently, their representations did not fully overlap and showed a noticeable separation in PCA space.

Figure S4. Rank-biased overlap (RBO) between fold-specific protein-level attention rankings compared with a matched random baseline, computed as described in the Supplementary Methods, *Case Study* section. For each of 1 000 drug-bacteria pairs sampled from the high-reliability main dataset, the top-k proteins defined by the ensemble (fold-averaged) Cross 2 attention ranking were compared across the four fold-specific rankings, and the resulting RBO scores are shown for the model (blue) and for a baseline in which the k proteins were instead drawn at random for each fold (orange), with kernel density estimates overlaid and dashed lines indicating distribution means. (A) Top 5% threshold (mean RBO: 0.399 vs. 0.073). (B) Top 10% threshold (mean RBO: 0.280 vs. 0.023). At both thresholds the model’s RBO substantially exceeded the random baseline, and the larger gap at the top 5% threshold indicates that top-ranked proteins carry a more reproducible relative ranking.

Figure S5. Attention-based visualization of drug-bacteria interactions for three case-study drugs. (A-F) Fold-ensembled atom-level attention maps for digoxin, sulfasalazine, and diltiazem, respectively, each shown for remaining top-ranked proteins belonging to the corresponding enriched EC classes. (A) WP_009306248.1 protein for digoxin. (B) WP_015760529.1 protein for digoxin. (C) WP_011107784.1 protein for diltiazem. (D) WP_011109241.1 protein for diltiazem. (E) WP_048696912.1 protein for diltiazem. (F) WP_002356161.1 protein for sulfasalazine. The highlighted reaction-relevant regions are the $\boldsymbol{\alpha}\mathbf{,}\boldsymbol{\beta}$-unsaturated lactone ring of digoxin, the azo bond of sulfasalazine, and the acetyloxy group of diltiazem.

**References**

[1]	RDKit: Open-Source Cheminformatics. https://www.rdkit.org

[2]	Olayo-Alarcon R, Amstalden MK, Zannoni A, Bajramovic M, Sharma CM, Brochado AR, Rezaei M, Müller CL. Pre-trained molecular representations enable antimicrobial discovery. Nature Communications. 2025;16(1):3420. doi:10.1038/s41467-025-58804-4

[3]	Dubchak I, Muchnik I, Holbrook SR, Kim SH. Prediction of protein folding class using global description of amino acid sequence. Proceedings of the National Academy of Sciences. 1995;92(19):8700–8704. doi:10.1073/pnas.92.19.8700

[4]	Mckenna A, Dubey S. Machine learning based predictive model for the analysis of sequence activity relationships using protein spectra and protein descriptors. Journal of Biomedical Informatics. 2022;128:104016. doi:10.1016/j.jbi.2022.104016

[5]	Adam M. protpy. (protpy - Package for generating protein physicochemical, biochemical and structural descriptors using their constituent amino acids). https://github.com/amckenna41/protPy?tab=readme-ov-file

[6]	Algavi YM, Borenstein E. A data-driven approach for predicting the impact of drugs on the human microbiome. Nature Communications. 2023;14(1):3614. doi:10.1038/s41467-023-39264-0

[7]	Ogata H, Goto S, Sato K, Fujibuchi W, Bono H, Kanehisa M. KEGG: Kyoto Encyclopedia of Genes and Genomes. Nucleic Acids Research. 1999;27(1):29–34. doi:10.1093/nar/27.1.29

[8]	Aramaki T, Blanc-Mathieu R, Endo H, Ohkubo K, Kanehisa M, Goto S, Ogata H. KofamKOALA: KEGG Ortholog assignment based on profile HMM and adaptive score threshold. Bioinformatics. 2020;36(7):2251–2252. doi:10.1093/bioinformatics/btz859

[9]	ESM Team. esm2024cambrian. 2024. (ESM Cambrian: Revealing the mysteries of proteins with unsupervised learning). https://evolutionaryscale.ai/blog/esm-cambrian

[10]	Hayes T, Rao R, Akin H, Sofroniew NJ, Oktay D, Lin Z, Verkuil R, Tran VQ, Deaton J, Wiggert M, et al. Simulating 500 million years of evolution with a language model. Science. 2025;387(6736):850–858. doi:10.1126/science.ads0018

[11]	Lin Z, Akin H, Rao R, Hie B, Zhu Z, Lu W, Smetanin N, Verkuil R, Kabeli O, Shmueli Y, et al. Evolutionary-scale prediction of atomic-level protein structure with a language model. Science. 2023;379(6637):1123–1130. doi:10.1126/science.ade2574

[12]	Leclercq M, Droit A. Protein Language Models: Applications and Perspectives. Journal of Proteome Research. 2026;25(2):507–524. doi:10.1021/acs.jproteome.5c00506

[13]	OECD (2023). (Q)SAR Assessment Framework: Guidance for the regulatory assessment of (Quantitative) Structure Activity Relationship models and predictions. doi:https://doi.org/10.1787/d96118f6-en

[14]	Jacoby C, Scorza K, Ecker L, Nol Bernardino P, Little AS, McMillin M, Ramaswamy R, Sundararajan A, Sidebottom AM, Lin H, et al. Gut bacteria metabolize natural and synthetic steroid hormones via the reductive OsrABC pathway. Cell Host & Microbe. 2025;33(11):1873-1885.e7. doi:10.1016/j.chom.2025.09.014

[15]	Mehta RS, Mayers JR, Zhang Y, Bhosle A, Glasser NR, Nguyen LH, Ma W, Bae S, Branck T, Song K, et al. Gut microbial metabolism of 5-ASA diminishes its clinical efficacy in inflammatory bowel disease. Nature Medicine. 2023;29(3):700–709. doi:10.1038/s41591-023-02217-7

[16]	Maini Rekdal V, Bess EN, Bisanz JE, Turnbaugh PJ, Balskus EP. Discovery and inhibition of an interspecies gut bacterial pathway for Levodopa metabolism. Science. 2019;364(6445):eaau6323. doi:10.1126/science.aau6323

[17]	Klünemann M, Andrejev S, Blasche S, Mateus A, Phapale P, Devendran S, Vappiani J, Simon B, Scott TA, Kafkia E, et al. Bioaccumulation of therapeutic drugs by human gut bacteria. Nature. 2021;597(7877):533–538. doi:10.1038/s41586-021-03891-8

[18]	Pedregosa F, Varoquaux G, Gramfort A, Michel V, Thirion B, Grisel O, Blondel M, Prettenhofer P, Weiss R, Dubourg V, et al. Scikit-learn: Machine Learning in Python. Journal of Machine Learning Research. 2011;12(85):2825–2830.

[19]	Newcombe RG. Two-sided confidence intervals for the single proportion: comparison of seven methods. Statistics in Medicine. 1998;17(8):857–872. doi:10.1002/(SICI)1097-0258(19980430)17:8<857::AID-SIM777>3.0.CO;2-E

[20]	Puza B, O’neill T. Generalised Clopper–Pearson confidence intervals for the binomial proportion. Journal of Statistical Computation and Simulation. 2006;76(6):489–508. doi:10.1080/10629360500107527

[21]	Wilson ID, Nicholson JK. Gut microbiome interactions with drug metabolism, efficacy, and toxicity. Microbiome and Human Disease Pathogenesis. 2017;179:204–222. doi:10.1016/j.trsl.2016.08.002

[22]	Peppercorn MA, Goldman P. The role of intestinal bacteria in the metabolism of salicylazosulfapyridine. J Pharmacol Exp Ther. 1972;181(3):555–562.

[23]	Fuller AT. IS p-AMINOBENZENESULPHONAMIDE THE ACTIVE AGENT IN PRONTOSIL THERAPY ? Originally published as Volume 1, Issue 5917. 1937;229(5917):194–198. doi:10.1016/S0140-6736(00)97447-6

[24]	Klotz U, Maier K, Fischer C, Heinkel K. Therapeutic efficacy of sulfasalazine and its metabolites in patients with ulcerative colitis and Crohn’s disease. New England Journal of Medicine. 1980;303(26):1499–1502.

[25]	Dobkin JF, Saha JR, Butler VP, Neu HC, Lindenbaum J. Digoxin-Inactivating Bacteria: Identification in Human Gut Flora. Science. 1983;220(4594):325–327. doi:10.1126/science.6836275

[26]	Luchi RJ, Gruber JW. Unusually large digitalis requirements: a study of altered digoxin metabolism. The American Journal of Medicine. 1968;45(2):322–328.

[27]	Zimmermann M, Zimmermann-Kogadeeva M, Wegmann R, Goodman AL. Mapping human microbiome drug metabolism by gut bacteria and their genes. Nature. 2019;570(7762):462–467. doi:10.1038/s41586-019-1291-3

[28]	Lindenbaum J, Rund DG, Butler Jr VP, Tse-Eng D, Saha JR. Inactivation of digoxin by the gut flora: reversal by antibiotic therapy. New England Journal of Medicine. 1981;305(14):789–794.

[29]	Weersma RK, Zhernakova A, Fu J. Interaction between drugs and the gut microbiome. Gut. 2020;69(8):1510. doi:10.1136/gutjnl-2019-320204

[30]	Wilson ID, Nicholson JK. Gut microbiome interactions with drug metabolism, efficacy, and toxicity. Transl Res. 2016;179:204–222. doi:10.1016/j.trsl.2016.08.002

[31]	Andre Martins, Ramon Astudillo. From Softmax to Sparsemax: A Sparse Model of Attention and Multi-Label Classification. In: Maria Florina Balcan, Kilian Q. Weinberger, editors. Proceedings of The 33rd International Conference on Machine Learning. Vol. 48. PMLR; 2016. p. 1614–1623. https://proceedings.mlr.press/v48/martins16.html

[32]	Peters B, Niculae V, Martins AFT. Sparse Sequence-to-Sequence Models. In: Korhonen A, Traum D, Màrquez L, editors. Proceedings of the 57th Annual Meeting of the Association for Computational Linguistics. Florence, Italy: Association for Computational Linguistics; 2019. p. 1504–1519. https://aclanthology.org/P19-1146/. doi:10.18653/v1/P19-1146

[33]	Jumper J, Evans R, Pritzel A, Green T, Figurnov M, Ronneberger O, Tunyasuvunakool K, Bates R, Žídek A, Potapenko A, et al. Highly accurate protein structure prediction with AlphaFold. Nature. 2021;596(7873):583–589. doi:10.1038/s41586-021-03819-2

[34]	Javdan B, Lopez JG, Chankhamjon P, Lee Y-CJ, Hull R, Wu Q, Wang X, Chatterjee S, Donia MS. Personalized Mapping of Drug Metabolism by the Human Gut Microbiome. Cell. 2020;181(7):1661-1679.e22. doi:10.1016/j.cell.2020.05.001
